# The UPR^mt^ preserves mitochondrial import to extend lifespan

**DOI:** 10.1083/jcb.202201071

**Published:** 2022-05-24

**Authors:** Nan Xin, Jenni Durieux, Chunxia Yang, Suzanne Wolff, Hyun-Eui Kim, Andrew Dillin

**Affiliations:** 1 Department of Molecular and Cell Biology, University of California, Berkeley, Berkeley, CA; 2 Howard Hughes Medical Institute, Chevy Chase, MD; 3 Department of Integrated Biology and Pharmacology, University of Texas, Health Science Center, Houston, TX

## Abstract

The mitochondrial unfolded protein response (UPR^mt^) is dedicated to promoting mitochondrial proteostasis and is linked to extreme longevity. The key regulator of this process is the transcription factor ATFS-1, which, upon UPR^mt^ activation, is excluded from the mitochondria and enters the nucleus to regulate UPR^mt^ genes. However, the repair proteins synthesized as a direct result of UPR^mt^ activation must be transported into damaged mitochondria that had previously excluded ATFS-1 owing to reduced import efficiency. To address this conundrum, we analyzed the role of the import machinery when the UPR^mt^ was induced. Using in vitro and in vivo analysis of mitochondrial proteins, we surprisingly find that mitochondrial import increases when the UPR^mt^ is activated in an ATFS-1–dependent manner, despite reduced mitochondrial membrane potential. The import machinery is upregulated, and an intact import machinery is essential for UPR^mt^-mediated lifespan extension. ATFS-1 has a weak mitochondrial targeting sequence (MTS), allowing for dynamic subcellular localization during the initial stages of UPR^mt^ activation.

## Teaser

UPR^mt^ increases mitochondrial quality control proteins and induces longevity. But how do the rescuing proteins enter mitochondria, as UPR^mt^ is initiated with dampened mitochondrial import? The study by Xin et al. shows that enhanced mitochondrial import mediates UPR^mt^-induced lifespan extension upon UPR^mt^ activation.

## Introduction

During the course of eukaryotic evolution and the development of sequestered organelles, communication events coevolved to allow proper homeostasis within the cell that encompassed master coordination by the nucleus. Such events have been discovered to include the unfolded protein response of the endoplasmic reticulum (UPR^ER^), the mitochondria (UPR^mt^), and the cytoplasmic heat shock response (Haynes and Ron, 2010; Kirstein-Miles and Morimoto, 2010; Liu and Chang, 2008). The primary mode of action of each of these stress pathways is the sensation of organelle-specific stress that is then communicated to the nucleus for transcriptional induction of the proper repair machinery to restore homeostasis within each organelle. Genes encoding compartment-specific chaperones are the sentinel transcriptional targets of such responses.

Mitochondria pose the most challenging organelle to coordinate stress responsiveness with the nucleus for several reasons. One, the mitochondrion is encapsulated within a double membrane system, the inner and outer membrane, thus creating a unique physical barrier that must be accommodated to signal from the mitochondrion to the nucleus. Two, the mitochondrion is composed of >1,000 different proteins, encoded within two distinct genomes, that comprise some of the largest complexes found in eukaryotic cells (Wiedemann and Pfanner, 2017; Hirst, 2013). Three, the byproducts of oxidative respiration, superoxide radicals, pose a constant challenge to mitochondrial integrity (Murphy, 2009). Four, import of proteins into the mitochondrion requires energy in the form of membrane potential and ATP created by the electron transport chain (Wiedemann and Pfanner, 2017), and each cell can contain hundreds of mitochondria that fuse and divide at any given time to change the cellular mitochondrial landscape. Taken together, monitoring the integrity of the mitochondrion and all of its various protein complexes with the ability to communicate to the nucleus to ensure the integrity of this organelle is extremely complex.

Mitochondria can orchestrate and coordinate a wide number of different stress-response mechanisms under various cellular and subcellular perturbations. Such responses include the UPR^mt^, mitophagy, and programmed cell death. In response to mild mitochondrial stress, the UPR^mt^, a specific transcriptional stress response system that is mediated by ATFS-1, DVE-1, UBL-5, LIN-65, MET-2, and PHF-8, is activated to increase the production of mitochondrial localized chaperones and proteases to help relieve the stress (Benedetti et al., 2006; Haynes et al., 2007; Merkwirth et al., 2016; Nargund et al., 2012; Tian et al., 2016). One of the major contributors to this response is ATFS-1. During mitochondrial stress, mitochondrial import efficiency is compromised, presumably due to depolarization of the mitochondrial membrane potential, which results in the inefficient import of the mitochondrial localized protein, ATFS-1. When ATFS-1 is not successfully imported into the mitochondria for degradation by mitochondrial proteases, it instead traffics to the nucleus, where it functions as a transcription factor, which coordinates with DVE-1, UBL-5, MET-2, and LIN-65 to induce the expression of mitochondrial chaperones and other genes required for repair of damaged mitochondria (Benedetti et al., 2006; Haynes et al., 2007; Merkwirth et al., 2016; Nargund et al., 2012; Tian et al., 2016). Inherent within this model is the balance that must be maintained between the membrane potential, import machinery, and the ability to induce the UPR^mt^. However, the link between membrane potential, mitochondrial import, and the UPR^mt^ is largely unexplored.

With the current model of ATFS-1 localization dynamics during stress exists a dichotomy in regard to induction of the UPR^mt^ and mitochondrial import. The repair proteins synthesized as a direct result of UPR^mt^ activation by ATFS-1 must be transported into damaged mitochondria that must have a depolarized membrane potential preventing the import of ATFS-1 into the mitochondria, providing entry of ATFS-1 into the nucleus. If ATFS-1 is unable to enter the mitochondria during stress, how then are other proteins allowed entry, especially damaged mitochondria with reduced membrane potential? Could there be coordination to increase mitochondrial import efficiency once the UPR^mt^ is induced? Is the mitochondrial import machinery a distinct branch of the UPR^mt^ to overcome the lack of integrity of damaged mitochondria? To address these questions, we analyzed the role of the import machinery under conditions where the UPR^mt^ was induced. Using in vitro biochemical assays of mitochondrial import and in vivo analysis of mitochondrially localized proteins, we find that the efficiency of mitochondrial import increases when the UPR^mt^ is activated. More surprisingly, the increased import due to UPR^mt^ induction occurs when the mitochondrial membrane potential is decreased. Finally, we find that the induction of the import machinery is essential for UPR^mt^-mediated lifespan extension, and ectopic induction of the UPR^mt^ preserves import into late life.

## Results

### Assessing mitochondrial import capacity from *Caenorhabditis elegans* mitochondria

**Figure 1.**
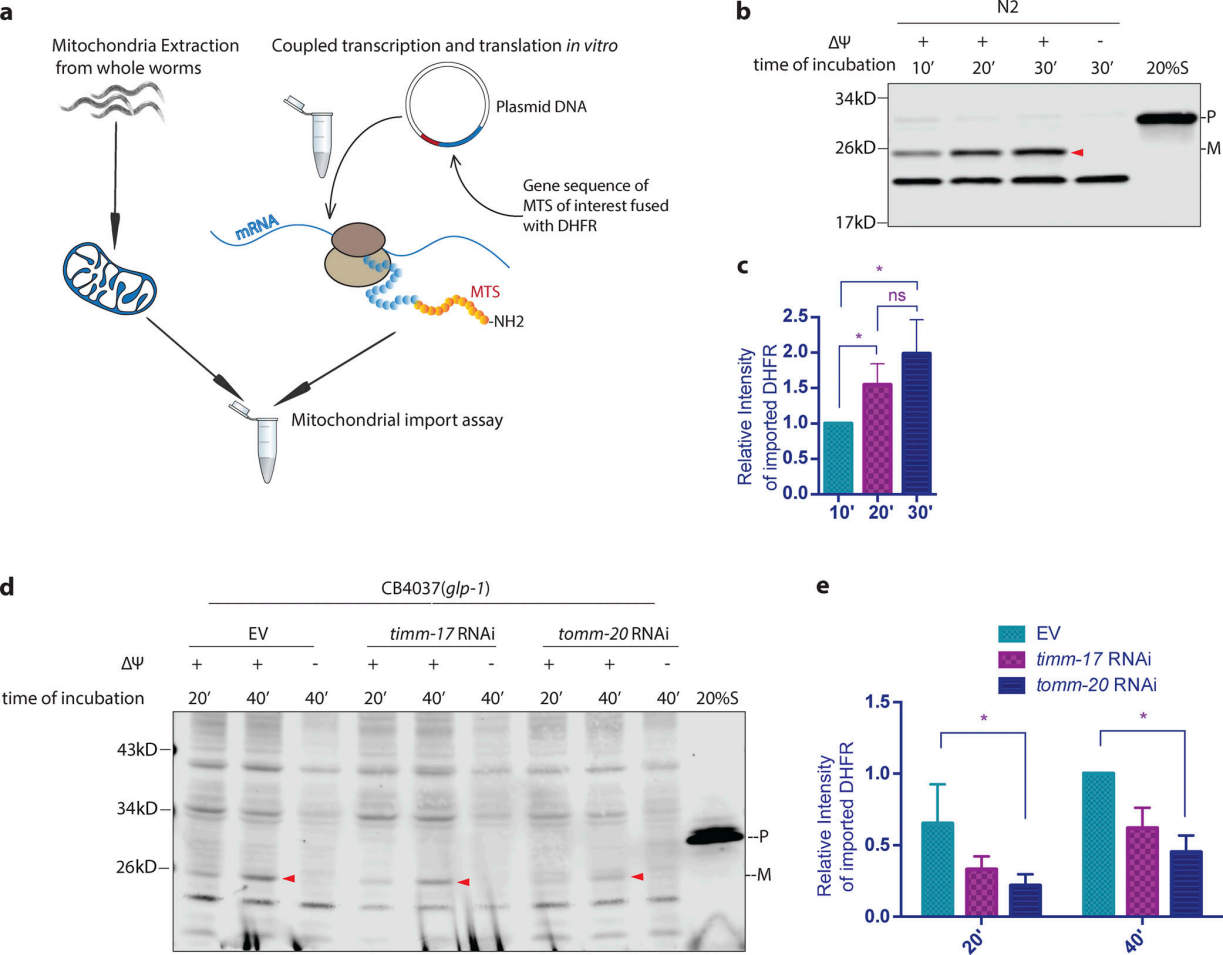
***C. elegans* mitochondrial protein in vitro import assay. (a)** Schematic diagram of the *C. elegans* in vitro mitochondrial protein import assay. **(b and c)** su9-DHFR was transcribed and translated in a single reaction with the Quick Coupled Transcription/Translation System (TnT reaction). Mitochondria extraction was made from synchronized N2 wild-type worms at day 1 of adulthood and quantified with BCA analysis. 50 µg mitochondrial protein was used in each reaction. The substrate protein was incubated with mitochondria extraction in import buffer containing an ATP regeneration system for 10, 20, or 30 min at 25°C. Mitochondria were subsequently treated with proteinase K to remove non-imported proteins. Upon being imported, the MTS of su9 is cleaved. 2 µg/ml valinomycin was used to disrupt the membrane potential (ΔΨ), thus inhibiting import. The precursor (p) and mature protein (m) were detected with the DHFR antibody by Western blot analysis. Right lane: 20% of the su9-DHFR substrate used in the import assay representing the precursor (p). **(d and e)** Germline-deficient, mutant *glp-1(e2141ts)* worms were bleach synchronized, grown at the restrictive temperature of 25°C, and treated with RNAi against *tomm-20* or *timm-17* until the first day of adulthood. Control worms were grown on bacteria containing empty vector alone. Mitochondria were isolated and subjected to the import assay. 2 µg/ml valinomycin was used to disrupt the membrane potential (ΔΨ), thus inhibiting import. **(c and e)** The efficiency of mitochondrial import was quantified by measuring the mature imported protein as detected by the DHFR antibody and analyzed with unpaired Student’s *t* test (two-tailed). All graphs are presented as mean ± SD. *, P < 0.05. *n* = 3 (c); *n* = 2 (e). Arrowheads, mature (imported) DHFR with the MTS cleaved off. Source data are available for this figure: SourceData F1.

All but a few mitochondrial proteins are transcribed from the nuclear genome and imported posttranslationally through the translocase of the outer/inner membrane (TOM/TIM) complex (Wiedemann et al., 2004; Wiedemann and Pfanner, 2017). Mitochondrial protein import depends on the mitochondrial membrane potential (ΔΨ), ATP, and is under the direction of mitochondrial targeting sequences (MTSs), which are cleaved after import (Chacinska et al., 2009). To measure the efficiency of mitochondrial import, we adapted and validated a method in which substrate proteins are synthesized in an in vitro transcription/translation reaction and subsequently imported into isolated mitochondria (Ryan et al., 2001; Stuart and Koehler, 2007; Fig. 1 a). We used a model import substrate, su9-DHFR, in which the MTS of subunit 9 of the mitochondrial ATP synthase from *Neurospora* is fused to a fragment of the cytosolic protein dihydrofolate reductase (DHFR) from mice (Pfanner et al., 1987). An ATP regeneration system (Khalimonchuk et al., 2006) was applied to increase the efficiency of import. Importantly, a DHFR antibody can readily detect the fusion protein being imported (Fig. 1, b and c), as indicated by (1) the change in size from a precursor protein to a mature DHFR; (2) the absence of mature DHFR upon disruption of membrane potential (ΔΨ); (3) the absence of precursor protein upon proteinase K treatment; and (4) the accumulation of a mature DHFR in a time-dependent manner. Mitochondrial import efficiency of su9-DHFR was reduced by 30–40% from mitochondrial preparations isolated from animals upon knocking down essential components of the TOM/TIM complex, *tomm-20* or *timm-17*, via RNAi, further validating the sensitivity and fidelity of the assay (Fig. 1, d and e).

### Mitochondrial import is enhanced upon UPR^mt^ induction

**Figure 2.**
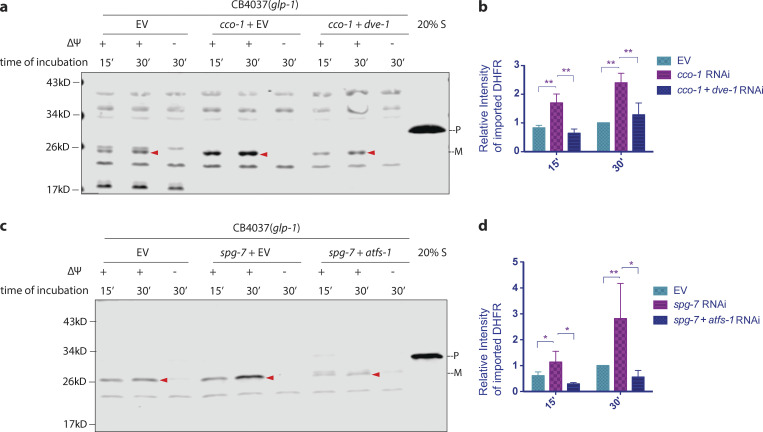
**The UPR**^**mt**^ **promotes mitochondrial import. (a–d)** To induce UPR^mt^ during development, *glp-1(e2141ts)* mutant animals were grown at 25°C on bacteria expressing *cco-1* dsRNA (a and b) or *spg-7* dsRNA (c and d) from the time of hatching until the first day of adulthood (animals were treated with 1:1 mixture of bacteria containing the empty RNAi vector alone [EV] to match with the double RNAi treatment). To suppress UPR^mt^, animals were treated with double RNAi (1:1 mixture of bacteria replacing EV with bacteria expressing *dve-1* dsRNA [a and b] or *atfs-1* dsRNA [c and d]). Control worms were grown on bacteria containing EV alone. Mitochondria were isolated from the animals at day 1 of adulthood and subjected to the import assay followed by Western blot analysis. Arrowheads, mature (imported) DHFR with the MTS cleaved off (a and c). Import efficiency was quantified by measuring the mature imported protein (arrowheads) as detected by the DHFR antibody, followed by analysis with unpaired Student’s *t* test (two-tailed; b and d). All graphs are presented as mean ± SD. *n* = 3. *, P < 0.05; **, P < 0.01. Source data are available for this figure: SourceData F2.

**Figure S1.**
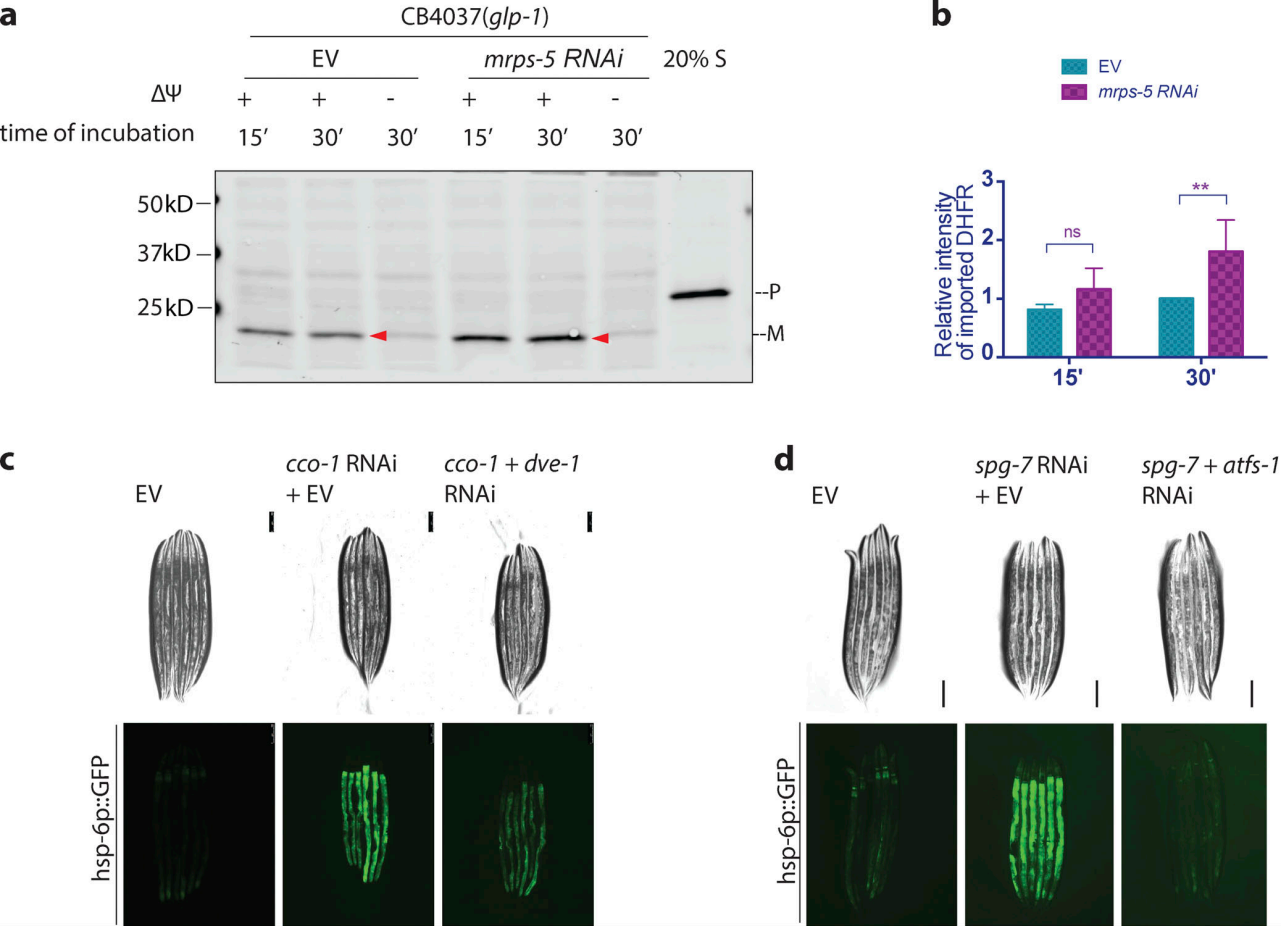
***mrps-5* knockdown promotes mitochondrial import. (a and b)** *glp-1(e2141ts*) mutant animals were grown at 25°C on bacteria expressing *mrps-5* dsRNA (20% diluted with bacteria containing the empty RNAi vector alone) from hatching until the first day of adulthood. Mitochondria were isolated on day 1 of adulthood and subjected to import assay followed by Western blot analysis. Import efficiency was quantified by measuring the mature imported protein (arrowheads) as detected by the DHFR antibody, followed by analysis with unpaired Student’s *t* test (two-tailed). The graph is presented as mean ± SD. *n* = 3. **, P < 0.01. **(c and d)** To induce UPR^mt^ during development, *hsp-6p*::*gfp* animals were grown at 25°C on bacteria expressing *cco-1* dsRNA (c) or spg-7 dsRNA (d; diluted to 1:1 ratio with bacteria containing the empty RNAi vector alone) from the time of hatching until the first day of adulthood. To suppress UPR^mt^, animals were treated with double RNAi (1:1 mixture of bacteria) replacing the empty RNAi vector with *dve-1* dsRNA (c) or *atfs-1* dsRNA (d). Control worms were grown on bacteria containing empty vector alone. Scale bar = 100 μm. Source data are available for this figure: SourceData FS1.

To understand whether and how mitochondrial import is regulated upon mitochondrial stress in somatic cells, where the UPR^mt^ has the greatest impact on longevity (Dillin et al., 2002), we induced the UPR^mt^ in *C. elegans* lacking their germline, the CB4037 *glp-1(e2141ts)* (Kodoyianni et al., 1992) sterile strain, and examined the import capacity of isolated mitochondria from these animals. We found that in mitochondria isolated from *glp-1(e2141ts)* mutant animals treated with *cco-1* RNAi, import capacity was elevated 2–2.5 times that of age-matched, mock RNAi control–treated animals (Fig. 2, a and b). More importantly, the enhanced import depends on the activation of the UPR^mt^. Loss of *dve-1*, a major transcription factor required for the UPR^mt^ (Fig. S1 c), resulted in marked suppression of import capacity that had been enhanced by *cco-1* knockdown (Fig. 2, a and b), indicating that the induction of the UPR^mt^ is essential for increased import efficiency.

Struck by the robust increase in mitochondrial import efficiency conferred by induction of the UPR^mt^ by *cco-1* RNAi in animals composed only of somatic cells, we asked whether the increased import efficiency was a common response to mitochondrial stress. SPG-7 is an AAA protease involved in quality control of mitochondrial membrane proteins, as well as the assembly of protein complexes on the mitochondrial inner membrane (Nolden et al., 2005). MRPS-5 is a mitochondrial ribosome protein (Houtkooper et al., 2013). Knockdown of either *spg-7* or *mrps-5*, via RNAi, induces the UPR^mt^ and leads to lifespan extension of worms composed of postmitotic, somatic cells. We found that mitochondrial import was also significantly enhanced upon either *spg-7* RNAi (Fig. 2, c and d) or *mrps-5* RNAi (Fig. S1, a and b). Furthermore, loss of ATFS-1, another major transcription factor required for UPR^mt^ induction (Fig. S1 d), suppressed the increased import of *spg-7* RNAi–treated animals (Fig. 2, c and d). In sum, induction of the UPR^mt^, by loss of *cco-1*, *mrps-5*, or *spg-7* resulted in robust increase of mitochondrial import that was dependent on either *dve-1* or *atfs-1*.

The in vitro analysis of mitochondrial import revealed increased import due to induction of the UPR^mt^. We sought to verify our in vitro results in vivo, but no in vivo mitochondrial import assays have been established in any eukaryote, let alone a metazoan. Therefore, we turned to in vivo analysis of endogenously localized mitochondrial proteins using immunoblot analysis, immunofluorescence, and enzymatic activity.

**Figure 3.**
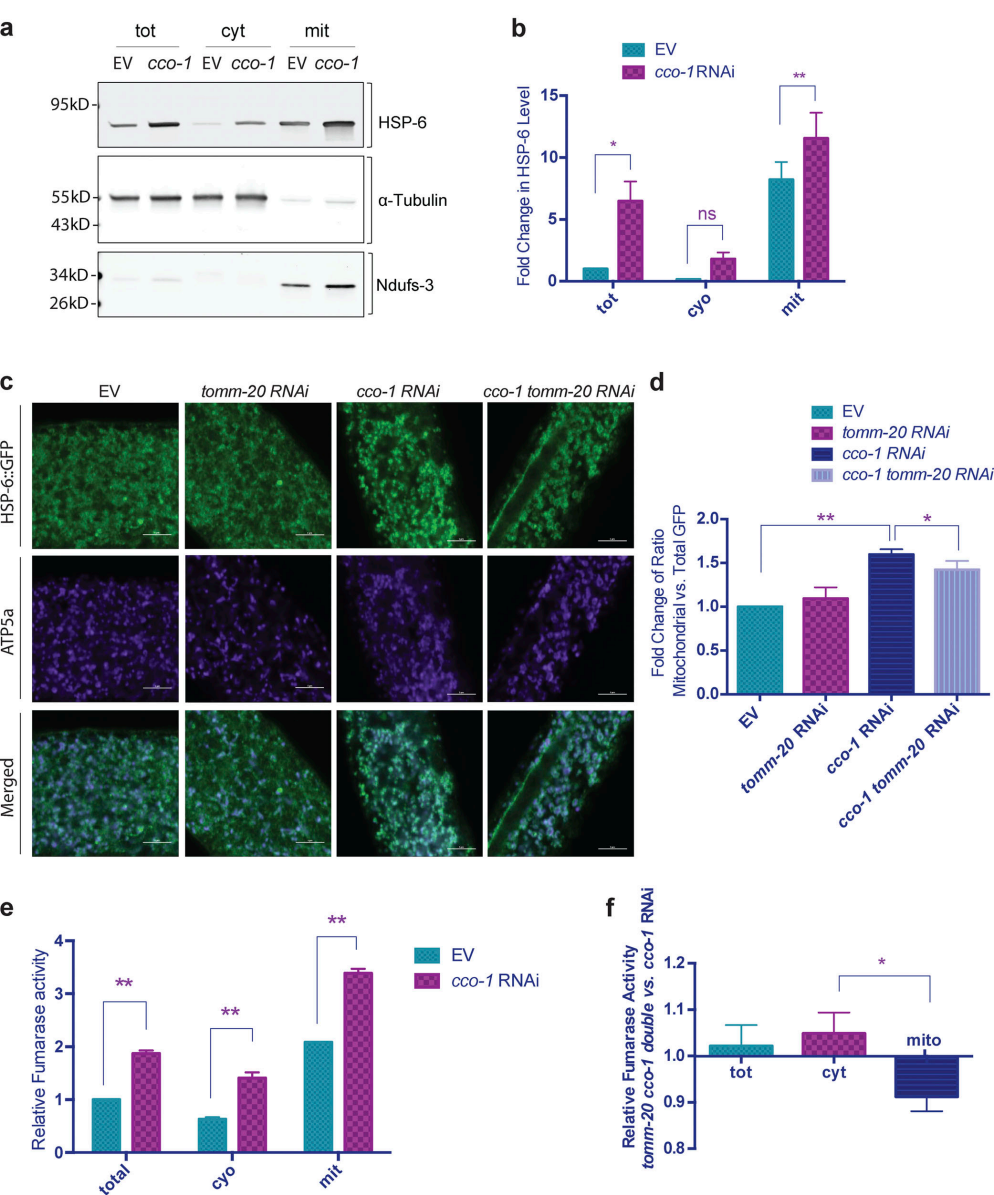
**The UPR**^**mt**^ **promotes mitochondrial import in vivo. (a and b)** Subcellular fractionation and Western blot of mitochondrial chaperone HSP-6. *glp-1* animals were grown at 25°C on bacteria expressing *cco-1* dsRNA from hatching until the first day of adulthood. Control worms were grown on bacteria containing the empty RNAi vector alone. Different fractions were separated via differential centrifugation. **(a)** Representative blot of HSP-6, α-tubulin, and Ndufs-3. **(b)** Signal intensity was normalized to total protein level (Ponceau S staining). HSP-6 level in each fraction was then normalized to that of the total fraction in the empty RNAi vector control. The graph is presented as mean ± SEM, *n* = 3. Ratio paired *t* test (two-tailed) was performed for each subcellular fraction to compare the difference between HSP-6 with and without the induction of UPR^mt^. *, P < 0.05; **, P < 0.01. **(c and d)** Immunostaining and confocal imaging of HSP-6p::HSP-6::GFP. **(c)** Representative images. Green, GFP; purple, ATP-5a. Scale bar = 5 μm. **(d)** Images were quantified with Fiji for total GFP and mitochondrial GFP (GFP within the boundary of mitochondrial marker ATP5a). The relative quantity of mitochondrial HSP-6::GFP was calculated as the ratio between mitochondrial and total GFP signals. This ratio for worms with different RNAi treatment was then normalized to the empty vector control. Mitochondrial HSP-6::GFP increased by 60% upon the induction of UPR^mt^ by *cco-1* RNAi. This increase was partially suppressed by compromising mitochondrial import capacity through knocking down *tomm-20.* The graph is presented as mean ± SEM of three biological repeats. For each condition, total number of worms imaged was *n* ≥ 8, total number of images quantified *n* ≥ 15. *t* test (two-tailed) was performed to compare the difference between different treatments. *, P < 0.05; **, P < 0.01. **(e and f)** Comparison of fumarase activity in different subcellular fractions. **(e)** Fumarase activity in each fraction was compared for animal with or without the induction UPR^mt^. Fumarase activity was normalized to the quantity of protein used in the assay. The graph is presented as mean ± SEM, *n* = 2. Paired *t* test (two-tailed) was performed for each subcellular fraction to compare the differences, **, P < 0.01. **(f)** Fumarase activity in each fraction was compared between *cco-1* knocked down animals treated with or without *tomm-20* RNAi. Histogram shows the changes of fumarase activity in each fraction, as represented by the ratio between animals treated with *tomm-20 cco-1* double RNAi and *cco-1* single RNAi (x–y intersection is set to [0,1] to better show the change in ratio). A mild yet consistent increase in cytosolic fraction and a corresponding decrease in the mitochondrial fraction were observed. The graph is presented as mean ± SEM, *n* = 3. Paired *t* test (two-tailed) was performed compare the difference between mitochondrial and cytosolic fractions. *, P < 0.05. Source data are available for this figure: SourceData F3.

**Figure S2.**
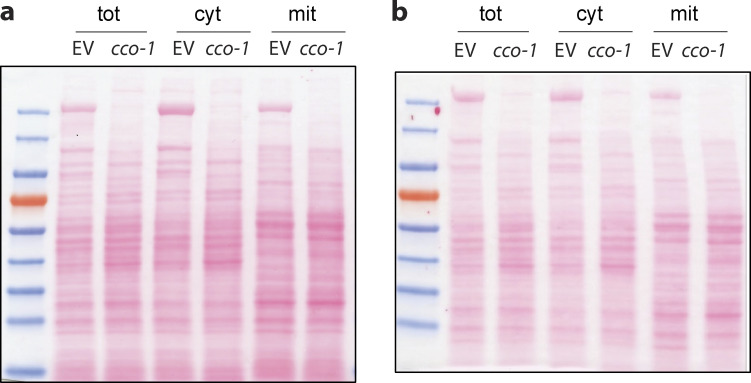
**Ponceau S staining shows protein at equivalent level. (a and b)** Representative Ponceau S staining of the Western blot of subcellular fractionation.

One, we analyzed the localization of endogenous HSP-6 via subcellular fractionation and Western blot. Upon UPR^mt^ activation, by *cco-1* RNAi, the level of endogenous HSP-6 increased more than five times in the total fraction (cytoplasm plus mitochondria; Figs. 3, a and b; and Fig. S2, a and b). With increased synthesis of HSP-6 protein, a moderate accumulation of HSP-6 was observed in the cytosol. However, in support of increased mitochondrial import due to UPR^mt^ induction, the majority of HSP-6 was found in the mitochondrial fraction.

Two, we generated a transgenic strain with single-copy insertion *hsp-6p::hsp-6::GFP*, allowing us to follow GFP localization as a function of HSP6 import. We induced the UPR^mt^ with *cco-1* RNAi and examined the localization of HSP-6::GFP in day 1 adults via immunofluorescent staining and confocal imaging. Upon the induction of UPR^mt^, the level of HSP-6::GFP increased to a similar level, as shown in our Western blot analysis (Fig. 3, c and d), and HSP-6::GFP colocalized with the mitochondrial marker ATP5a, suggesting that the increasingly synthesized HSP-6 was imported into the mitochondria. Likewise, knocking down a major TOM complex component, *tomm-20,* decreased the mitochondrial localization of HSP-6::GFP, suggesting that an intact import machinery is required for the mitochondrial localization of HSP-6 when the UPR^mt^ is induced.

Three, we examined the mitochondrial import of fumarase, a dually localized enzyme in both mitochondria and cytoplasm. The localization of fumarase is subject to the regulation of mitochondrial import (Yogev et al., 2010). When the UPR^mt^ was induced with *cco-1* RNAi, we found a mild induction of total fumarase activity in both the cytoplasm and the mitochondria (Fig. 3 e). When *tomm-20* was knocked down during the induction of UPR^mt^, we found a consistent decrease of the fumarase activity in the mitochondrial fraction and increased activity in the cytosolic fraction (Fig. 3 f).

In summary, UPR^mt^ induction by loss of *cco-1*, *spg-7*, or *mrps-5* resulted in increased mitochondrial import using our in vitro assay that was dependent on either *dve-1* or *atfs-1*. Furthermore, in vivo analysis, following either endogenous levels of HSP-6 by subcellular fractions or GFP-tagging and immunofluorescence, finds HSP-6 preferentially in the mitochondria in a *tomm-20*–dependent manner. Lastly, following the enzymatic activity of dual localized enzyme, fumarase, we find more activity in the mitochondria upon UPR^mt^ induction, also *tomm-20* dependent. Therefore, turning on the UPR^mt^ increases mitochondrial import.

### Mitochondrial import machinery is upregulated upon UPR^mt^ induction

**Figure 4.**
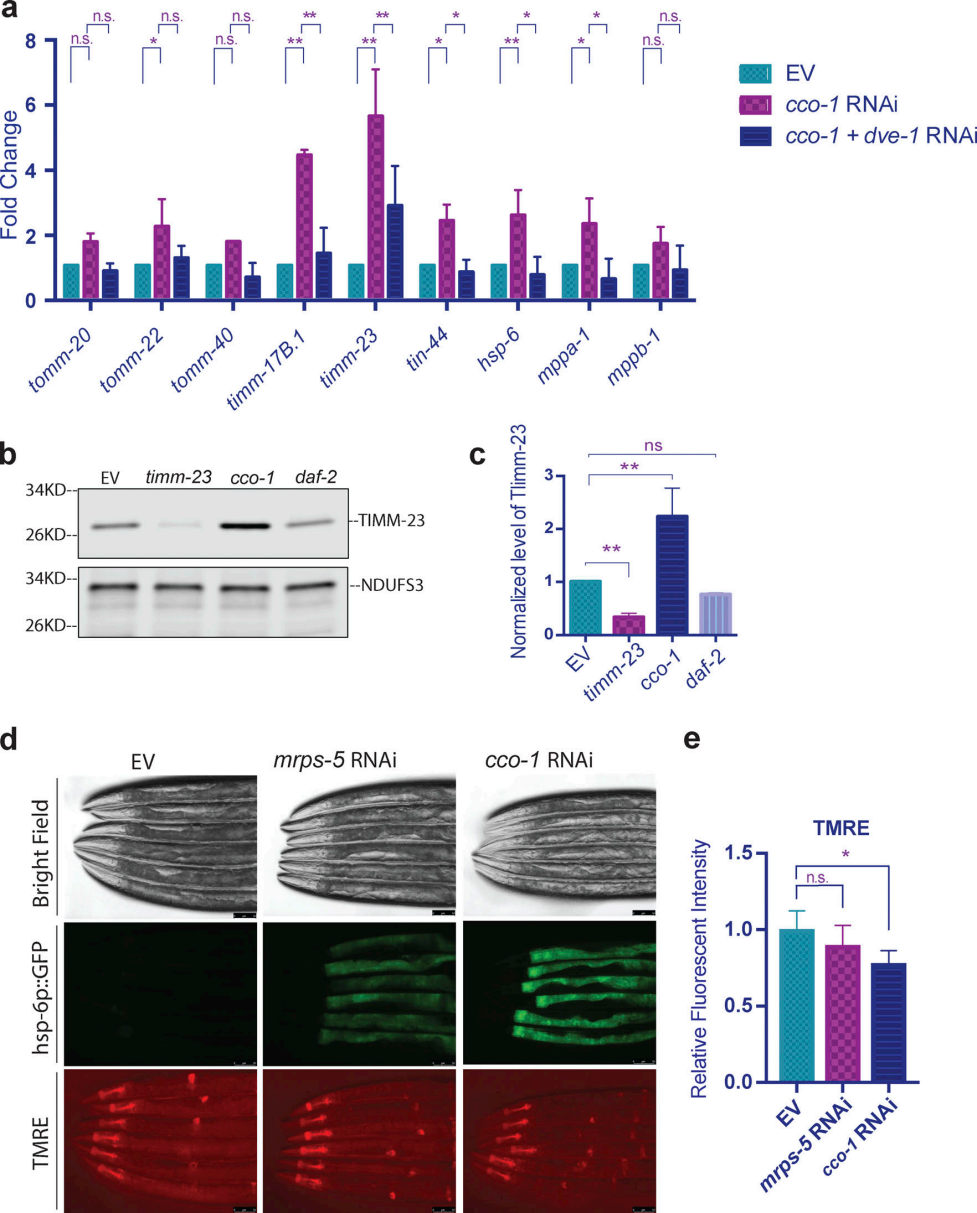
**The UPR**^**mt**^ **enhances the transcription of mitochondrial import machinery. (a)** To induce UPR^mt^ during development, *glp-1(e2141ts*) mutant animals were grown at 25°C on bacteria expressing *cco-1* dsRNA from the time of hatching until the first day of adulthood (animals were treated with 1:1 mixture of bacteria containing the empty RNAi vector alone [EV] to match with the double RNAi treatment). To suppress UPR^mt^, animals were treated with double RNAi (1:1 mixture of bacteria replacing EV with bacteria expressing *dve-1* dsRNA). Control worms were grown on bacteria containing EV alone. RNA was isolated on day 1 of adulthood, and qPCR analysis was performed. Expression was normalized against three housekeeping genes and quantified with Student’s *t* test (two-tailed). The graph is presented as mean ± SD, *n* = 2. *, P < 0.05; **, P < 0.01. **(b and c)** *glp-1* animals were grown at 25°C on bacteria expressing *timm-23*, *cco-1*, or *daf-2* dsRNA (each was diluted to 1:1 ratio with bacteria containing the empty RNAi vector alone) from hatching until the first day of adulthood. Control worms were grown on bacteria containing the empty vector alone. Quantification is shown in c with unpaired Student’s *t* test (two-tailed). *n* = 3. **, P < 0.01. The signal intensity of TIMM-23 was normalized to that of NDUFS3. **(d and e)** *hsp-6p*::*gfp* animals were grown at 20°C and treated with RNAi or empty RNAi vector control from hatching until the L4 stage, then transferred to plates of the same RNAi treatment with the addition of TMRE and grown overnight. Bacteria expressing *cco-1* dsRNA (right) or *mrps-5* dsRNA (middle; both were diluted 20% with bacteria containing the empty RNAi vector alone) were used to induced UPR^mt^. Control worms were grown on bacteria containing the RNAi vector alone (left). Scale bar = 50 μm. Fluorescent intensity was quantified with ImageJ and analyzed with unpaired Student’s *t* test (two-tailed). *n* = 3. *, P < 0.05. All graphs are presented as mean ± SD. Source data are available for this figure: SourceData F4.

**Figure S3.**
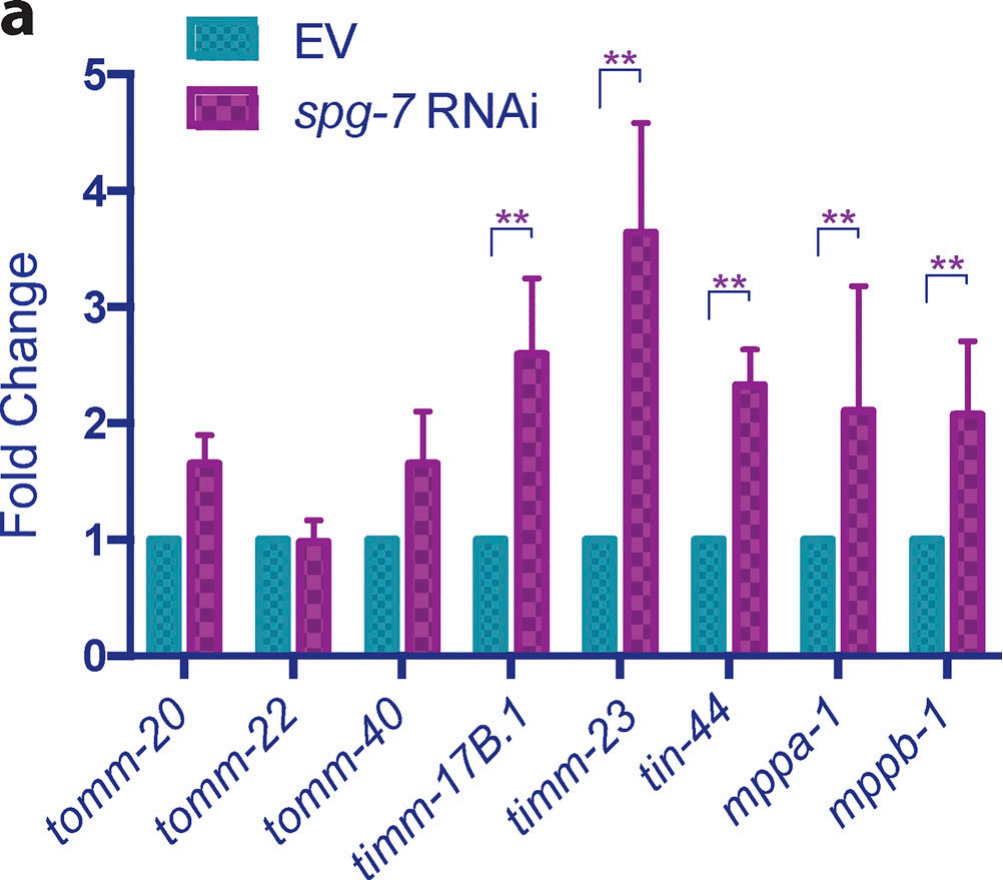
***spg-7* knockdown enhances the transcription of import machinery components. (a)** *glp-1(e2141ts)* animals were grown at 25°C on bacteria expressing *spg-7* dsRNA (diluted to 1:1 ratio with bacteria containing the empty RNAi vector alone) from hatching until the first of adulthood. RNA was isolated on day 1 of adulthood, and qPCR analysis was performed. Expression was normalized against three housekeeping genes and quantified with Student’s *t* test (two-tailed). The graph is presented as mean ± SD, *n* = 3. *, P < 0.05; **, P < 0.01.

The UPR^mt^ promotes mitochondrial protein homeostasis through signaling to the nucleus to induce the transcription of mitochondrial localized stress-responsive genes. Most of the protein products of the upregulated genes must then be imported into mitochondria to restore proteostasis. The proteins that constitute the import machinery, TIM/TOM complexes, are exclusively encoded by nuclear genes. Therefore, it is conceivable that the UPR^mt^ may enhance import through upregulating the expression of the TIM/TOM complex components. To examine the transcriptional regulation of import machinery in somatic tissue, we tested germline-deficient *glp-1(e2141ts)* mutant animals for the transcription of a series of genes encoding the mitochondrial import machinery. Comparing the synchronized and age-matched worms, we found that transcription of the TIM/TOM genes upon *cco-1* RNAi treatment was consistently higher than the mock RNAi control animals. Genes encoding TOM complex proteins, including *tomm-20*, *tomm-22*, and *tomm-40*, were enhanced one- to twofold. Core components of the TIM complex, *timm-17* and *timm-23*, were upregulated three- and sevenfold, respectively (Fig. 4 a). The transcription level of *timm-17* and *timm-23* at steady state appeared to be lower than other TIM/TOM components, whereas their transcription was elevated to levels higher than other components upon UPR^mt^ activation. This is consistent with previous findings, which suggests that TIM23 protein might be the rate-limiting factor in mitochondrial import (Donzeau et al., 2000). After passing through the inner membrane pore formed by TIM17 and TIM23, the precursor proteins are pulled into the matrix by TIM44 and mtHSP70 (HSP-6). mtHSP70 also facilitates the proper folding of imported proteins, which are subsequently processed by MPP proteins, proteases that cleave off the MTSs (Gakh et al., 2002). We found that *hsp-6* and *tin-44*, as well as *mppa-1* and *mppb-1*, which are *C. elegans* homologs of mammalian *mtHsp70*, *tim44*, and *Mpp*, respectively, were also upregulated by *cco-1* RNAi. Therefore, the entire repertoire of the import machinery appears to be transcriptionally induced by activation of the UPR^mt^ in somatic cells. Similarly, the TIM/TOM import machinery was also upregulated upon *spg-7* RNAi (Fig. S3 a). Additionally, the *cco-1*–induced upregulation of import machinery genes was suppressed by RNAi against *dve-1* (Fig. 4 a).

To further verify the regulation of import machinery, we generated an antibody against the *C. elegans* mitochondrial translocase protein TIMM-23 and verified its specificity with RNAi knockdown of *timm-23*. When comparing the endogenous level of TIMM-23, we found that the TIMM-23 protein level is indeed enhanced when the UPR^mt^ is activated (Fig. 4, b and c).

Given that mitochondrial protein import requires membrane potential (ΔΨ), we examined whether ΔΨ is enhanced upon UPRmt activation. Surprisingly, we did not observe stronger tetramethylrhodamine ethyl ester (TMRE) staining, a marker of ΔΨ, in worms with activated UPR^mt^ (Fig. 4, d and e). In contrast, membrane potential was found to be reversely correlated with the activation of the UPR^mt^. In particular, knockdown of *cco-1* induced the UPR^mt^ more robustly than knockdown of *mrps-5*, as indicated by the level of *hsp-6p::gfp* reporter, whereas the mitochondrial membrane potential is lower with *cco-1* knockdown compared with *mrps-5* knockdown (Fig. 4, d and e). Together, these findings suggest that the enhancement of mitochondrial import we observed in the in vitro import assay is regulated through increased transcriptional regulation of import machinery, rather than enhanced membrane potential. Furthermore, and most surprisingly, these results indicate that aspects of mitochondrial import can be decoupled from membrane potential.

### The MTS of ATFS-1 is less import competent

**Figure 5.**
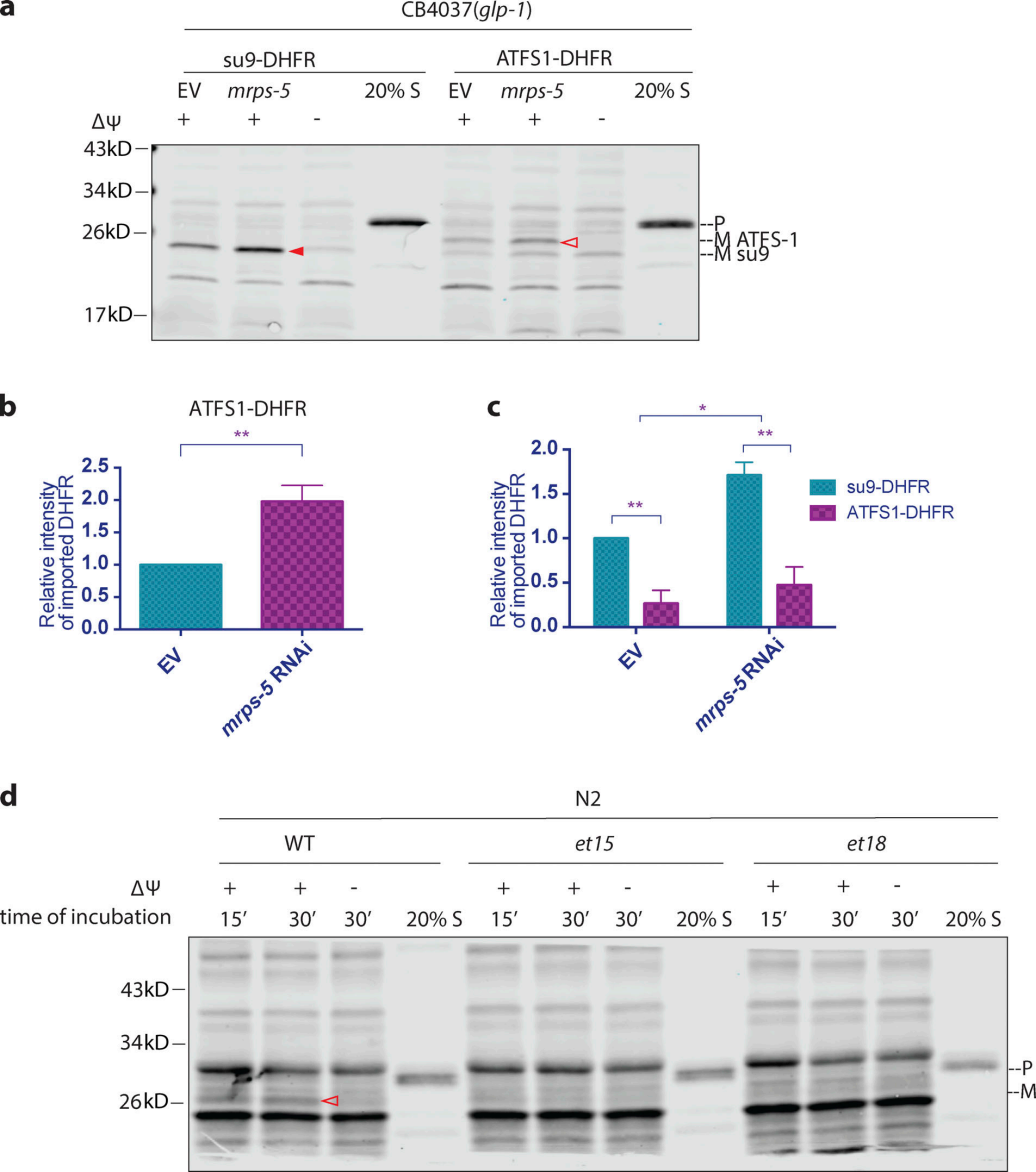
**The MTS of ATFS-1 is less import competent. (a–c)** Comparison of import competency between su9-DHFR and ATFS-1-DHFR. The N-terminus 73 amino acids of ATFS-1 was used as MTS. *glp-1(e2141ts*) animals were grown at 25°C on bacteria expressing *mrps-5* dsRNA (20% diluted with bacteria containing the empty RNAi vector alone) from hatching until the first day of adulthood. Mitochondria were isolated on day 1 of adulthood and subjected to import assay with 30-min incubation time, followed by Western blot analysis. Import efficiency was quantified by measuring the mature imported protein as detected by the DHFR antibody. Quantification of imported DHFR is shown in b and c. **(b)** Import of ATFS1-DHFR was compared between *mrps-5* RNAi and empty vector control with unpaired Student’s *t* test (two-tailed). The graph is presented as mean ± SD, *n* = 4. **, P < 0.01. **(c)** Import of DHFR with the MTS of su9 and ATFS-1 were compared with Student’s *t* test (two-tailed). The graph is presented as mean ± SD, *n* = 3. *, P < 0.05; **, P < 0.01. **(d)** Mitochondrial targeting capacity abolished by point mutations *et15* or *et18* in the MTS of ATFS-1. Mitochondria extraction was made from synchronized N2 wild-type worms at day 1 of adulthood and subjected to import assay with different substrates. Import with *et15* or *et18* was below the detectable level. Solid arrowheads, mature (imported) su9-DHFR with the su-9 MTS cleaved off; open arrowheads, mature (imported) ATFS1-DHFR with the MTS of ATFS-1 cleaved off. Source data are available for this figure: SourceData F5.

Presumably, the enhancement of import would allow efficient translocation of the repair proteins into the mitochondria to restore mitochondrial function. However, previous findings suggest that import is compromised upon mitochondrial stress, thereby allowing ATFS-1 to translocate into the nucleus, where it activates the transcription of stress response genes (Haynes and Ron, 2010). Is the import of ATFS-1 differentially regulated? To interrogate this possibility, we made a chimeric protein with the predicted MTS of ATFS-1 (N-terminal 73 amino acids; Nargund et al., 2012) fused with DHFR (ATFS1-DHFR). We found that upon the induction of the UPR^mt^, the import of ATFS1-DHFR is also upregulated (Fig. 5, a and b). Although the regulation of ATFS-1 import shows the same trend, we observed that the import of ATFS1-DHFR is less robust compared with su9-DHFR (Fig. 3 a). Indeed, when we compared the import of the two fusion proteins side by side, we found that mitochondrial import directed by the MTS of ATFS-1 was significantly less robust (Fig. 5, a and c). Our finding is consistent with the model that ATFS-1 has a weak MTS, which allows it to sense modest mitochondrial dysfunction and membrane depolarization to send a stress signal to the nucleus by translocation (Rolland et al., 2019; Melber and Haynes, 2018). Point mutations in the MTS of *atfs-1*, such as *et15* or *et18*, display constitutively active UPR^mt^ (Rauthan et al., 2013), presumably due to lack of mitochondrial import of ATFS-1 and relocation to the nucleus. We introduced the *et15* and *et18* mutations into the MTS of the ATFS1-DHFR construct and used them in the in vitro import assay. We found that the mitochondrial targeting capacity deteriorated with both mutations in the MTS of *atfs-1* (Fig. 5 d).

### Induction of the UPR^mt^ maintains mitochondrial import during aging

**Figure 6.**
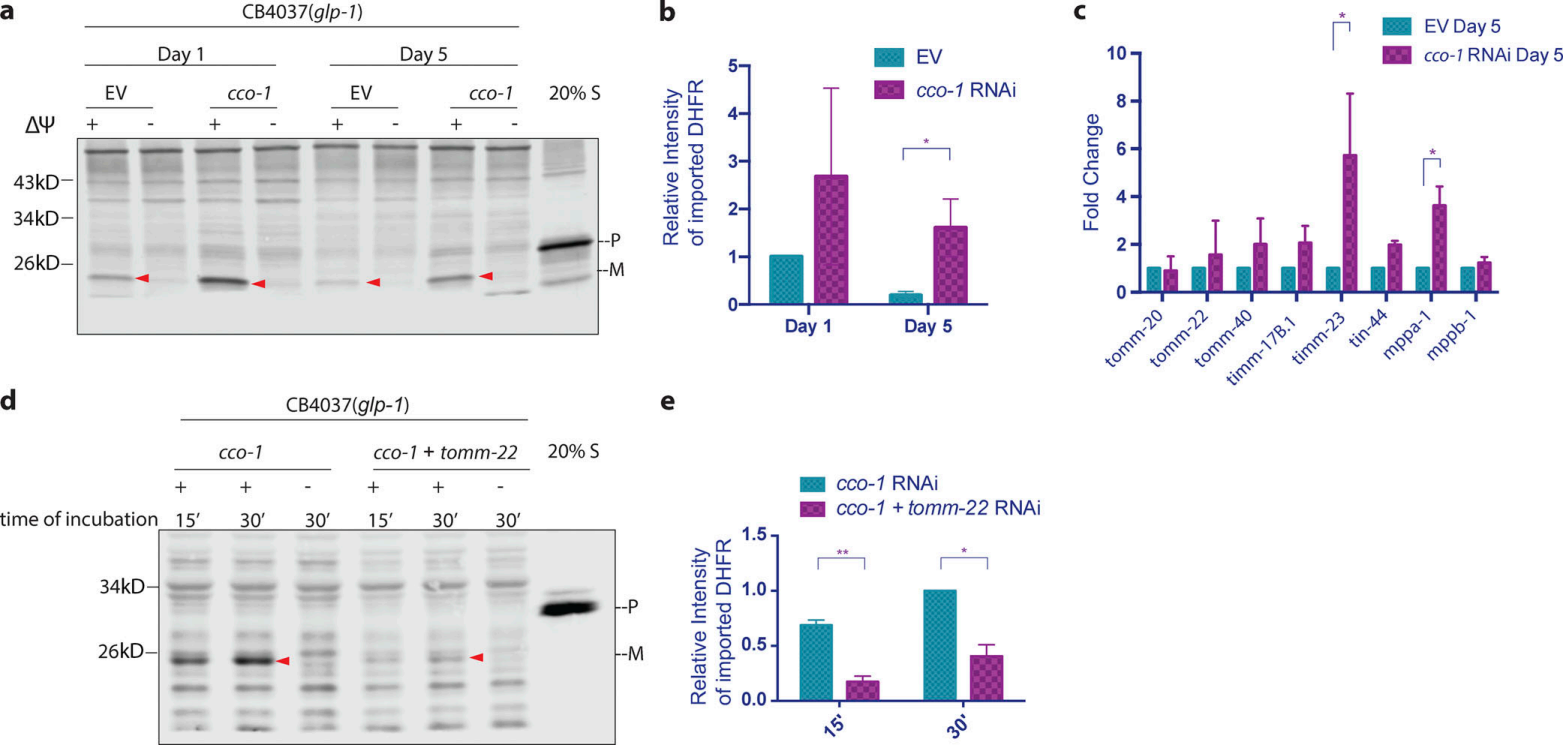
**UPR**^**mt**^ **delayed the age-associated decline of import. (a and b)** *glp-1(e2141ts*) worms were synchronized in two batches and grown at 25°C. When the two batches reached day 1 and 5 of adulthood, respectively, mitochondria were isolated in parallel and subjected to import assay with 30-min incubation time followed by Western blot analysis. Import efficiency was quantified by measuring the mature imported protein as detected by the DHFR antibody, followed by analysis with unpaired Student’s *t* test (two-tailed; b). Graph is presented as mean ± SD, *n* = 4. *, P < 0.05. Arrowheads, mature (imported) DHFR with the MTS cleaved off. **(c)** *glp-1(e2141ts)* worms were synchronized and grown at 25°C on bacteria expressing dsRNA from the time of hatching. RNA was isolated on day 5 of adulthood, and qPCR analysis was performed. Expression was normalized against three housekeeping genes. The graph is presented as mean ± SD, *n* = 2. *, P < 0.05. **(d and e)** *glp-1(e2141ts)* worms were synchronized and grown at 25°C on bacteria expressing dsRNA from the time of hatching. Mitochondria were isolated on day 1 of adulthood and subjected to import assay followed by Western blot analysis. Import efficiency was quantified by measuring the mature imported protein as detected by DHFR antibody, followed by analysis with unpaired Student’s *t* test (two-tailed; e). The graph is presented as mean ± SD, *n* = 2. *, P < 0.05; **, P < 0.01. Source data are available for this figure: SourceData F6.

Intrigued by the finding that enhanced import efficiency was required for UPR^mt^-mediated longevity, we asked how import efficiency might play a role in normal aging. As organisms age mitochondria gradually depolarize and the membrane potential, the major driving force of import, declines (Parihar and Brewer, 2007). We tested import efficiency across mitochondria isolated from aging cohorts of *C. elegans*. Indeed, we confirmed that mitochondrial import declines dramatically as the animals age (Fig. 6, a and b). Two age groups (days 1 and 5) were chosen to represent worms in the process of aging. We observed a more than one-half reduction in the import efficiency of su9-DHFR from day 1 to day 5. This finding suggests a catastrophic loss in import capacity early in the aging process.

Treating worms with RNAi against *cco-1* delayed the age-associated decline of import. For example, import remained significant on day 5 in *cco-1* RNAi–treated worms, whereas import in mock-RNAi control worms was barely detectable (Fig. 6, a and b). The transcriptional level of TIM/TOM import machinery also remained higher in older worms under *cco-1* RNAi treatment (Fig. 6 c).

**Figure S4.**
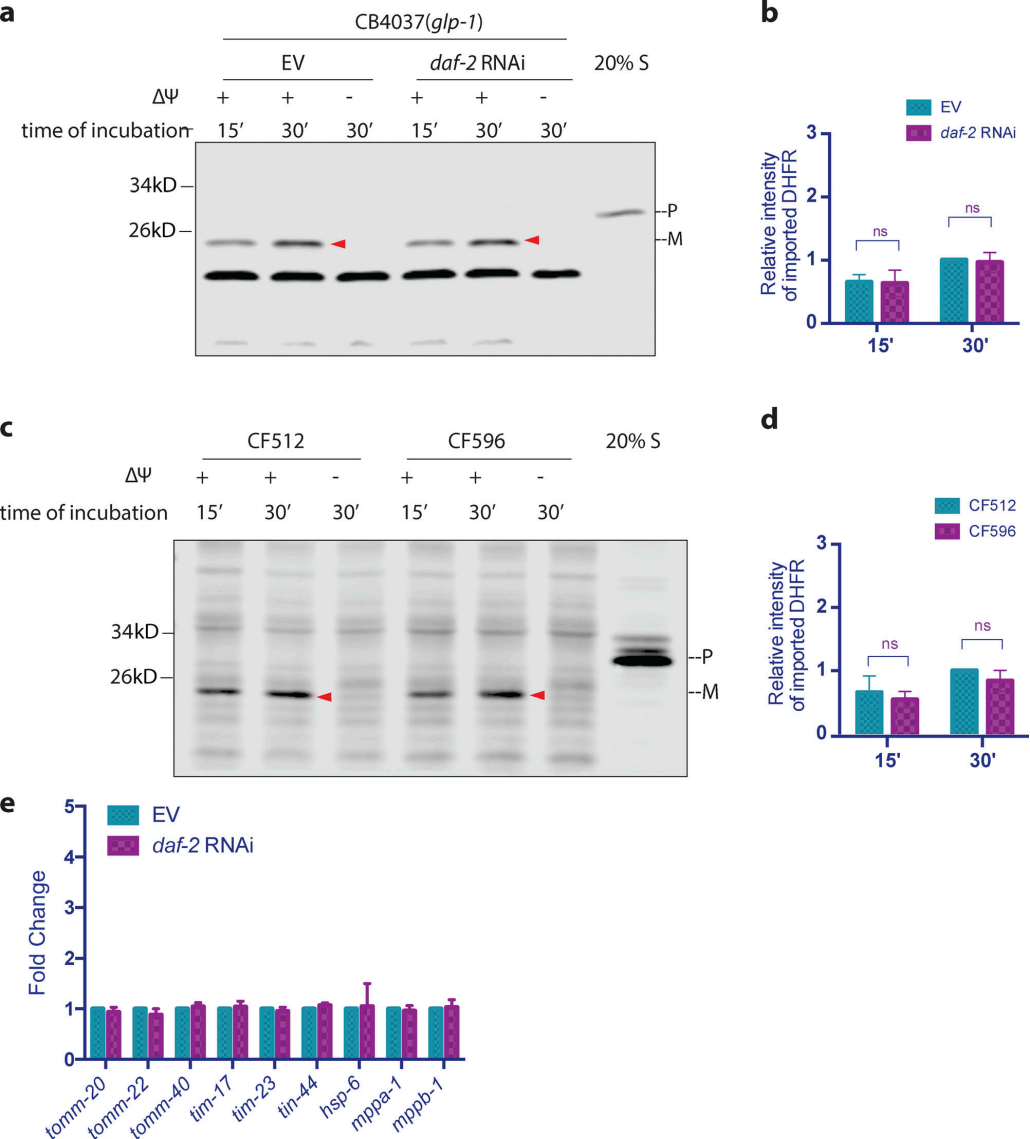
***daf-2* mutation or *daf-2* knockdown does not enhance mitochondrial import. (a and b)** *glp-1(e2141ts*) animals were grown at 25°C on bacteria expressing *daf-2* dsRNA (diluted to 1:1 ratio with bacteria containing the empty RNAi vector alone) from hatching until the first day of adulthood. Mitochondria were isolated on day 1 of adulthood and subjected to import assay followed by Western blot analysis. Graph is presented as mean ± SD, *n* = 3. **(c and d)** CF512 *fer-15(b26) II; fem-1(hc17ts) I* and CF596 *daf-2(mu150) III; fer-15(b26); fem-1(hc17ts)* worms were grown at 20°C until larval stage L2 and then transferred to 25°C. Mitochondria were isolated on day 1 of adulthood and subjected to import assay followed by Western blot analysis. Import efficiency was quantified by measuring the mature imported protein as detected by the DHFR antibody, followed by analysis with unpaired Student’s *t* test (two-tailed; b and d). Graph is presented as mean ± SD, *n* = 3. Arrowheads: mature (imported) DHFR with the MTS cleaved off. **(e)** *glp-1(e2141ts)* animals were grown at 25°C on bacteria expressing daf-2 dsRNA (diluted to 1:1 ratio with bacteria containing the empty RNAi vector alone) from hatching until the first of adulthood. RNA was isolated on day 1 of adulthood, and qPCR analysis was performed. Expression was normalized against three housekeeping genes and quantified with Student’s *t* test (two-tailed). The graph is presented as mean ± SD, *n* = 4. Source data are available for this figure: SourceData FS4.

As lifespan extension is a common effect of UPR^mt^ activation, it is intriguing to know whether the enhancement in mitochondrial import capacity we observed is a secondary effect of delayed aging or more specific to UPR^mt^ induced forms of longevity. We tested another major pathway that regulates lifespan, the insulin/IGF1 signaling pathway mediated by the insulin/IGF1 receptor, *daf-2* in worms. We found that import capacity in day 1 adult worms was not affected by either *daf-2* RNAi or the *daf-2(e1370)* mutation (Fig. S4, a–d), despite the dramatic increase in lifespan in these strains. In addition, upon knockdown of *daf-2*, we did not observe an increase in import machinery, at either the transcription level (Fig. S4 e) or protein level (Fig. 4, b and c). Taken together, these findings indicate that the enhancement of mitochondrial import in somatic cells is unique to UPR^mt^ activation, and the aging process itself reduces import that can be combated by UPR^mt^ activation.

### UPR^mt^-induced lifespan extension requires intact TIM/TOM complexes

Our findings suggest that the mitochondrial import machinery is upregulated at the transcriptional level upon UPR^mt^ induction, thereby elevating import capacity and allowing stress-responsive proteins to translocate into the mitochondrial matrix and restore proteostasis. Indeed, we found that an intact import machinery is required for the enhancement of import upon UPR^mt^ activation, as knocking down TOM complex component *tomm-22* abolished the effect of *cco-1* RNAi on improving import capacity (Fig. 6, d and e).

**Figure 7.**
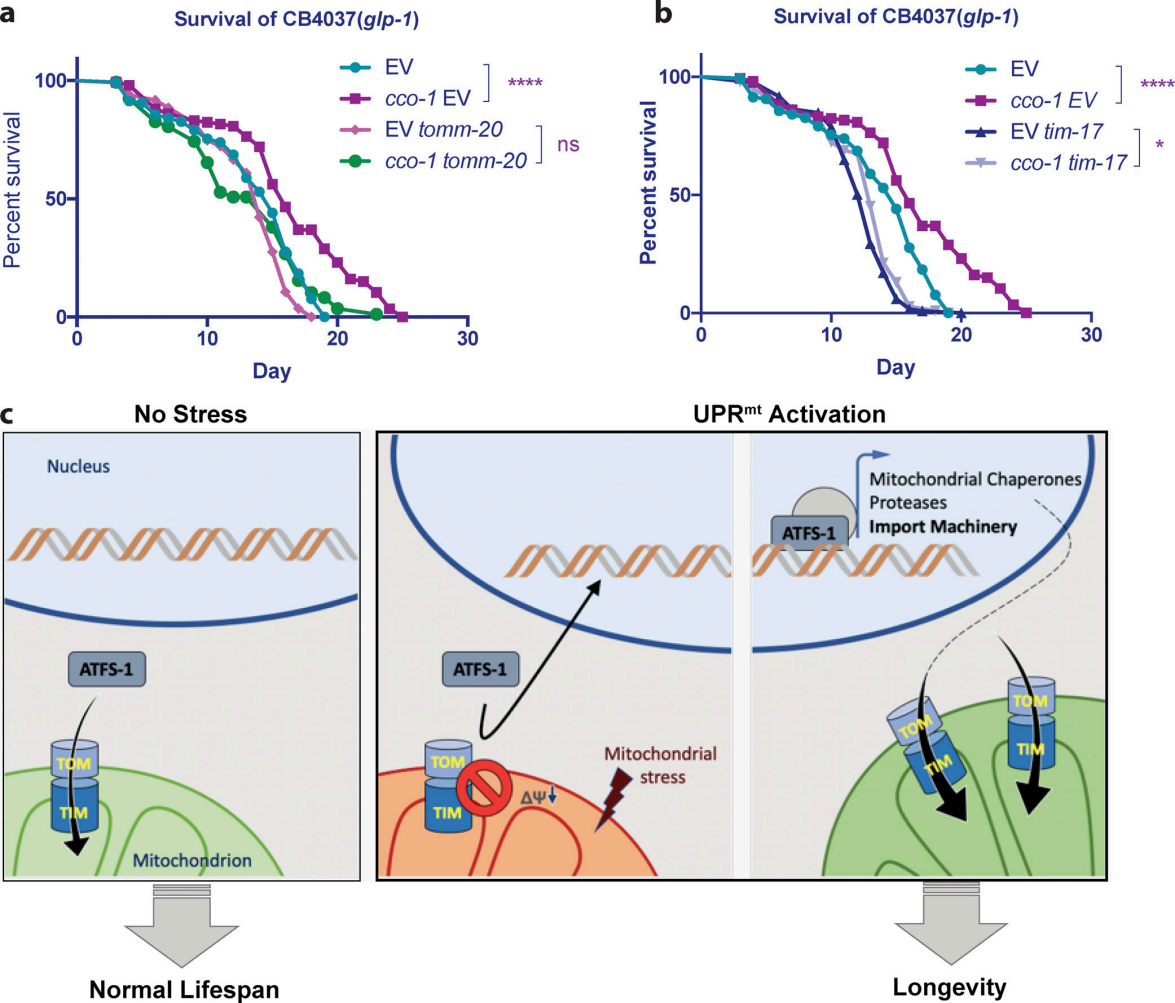
**Import machinery is required for UPR**^**mt**^**-dependent lifespan extension. (a and b)** Adult lifespans of *glp-1(e2141ts*) animals grown on bacteria expressing dsRNA from the time of hatching. Turquoise lines, the lifespan of animals grown on control bacteria containing the RNAi vector alone; purple lines, the lifespan of animals grown on bacteria expressing the dsRNA of *cco-1*. **(a)** Magenta lines, the lifespan of animals grown on bacteria expressing the dsRNA of *tomm-20*; green lines: the lifespan of animals grown on bacteria expressing the dsRNA of *cco-1* and the dsRNA of *tomm-20*. **(b)** Lavender blue lines, the lifespan of animals grown on bacteria expressing the dsRNA of *cco-1* and the dsRNA of *tim-17*; dark blue lines, the lifespan of animals grown on bacteria expressing the dsRNA of *tim-17*. a and b are presented separately for clarity. Log-rank (Mantel–Cox) method was used to determine the significant differences (*, P < 0.05; ****, P < 0.0001). **(c)** A schematic figure depicting the model of UPR^mt^-induced upregulation of mitochondrial import.

The next question we asked was whether the improvement of import is a necessary element for UPR^mt^-induced longevity. To test this, we used double RNAi to knockdown import activity in long-lived *cco-1*–deficient animals. We found that when worms were treated with *tomm-20* RNAi simultaneously with *cco-1* RNAi, the extension of lifespan was largely suppressed, whereas *tomm-20* RNAi had a minimal effect on lifespan in wild-type animals (Fig. 7 a). Similarly, treating worms with *timm-17* RNAi also partially suppressed the lifespan extension (Fig. 7 b). These results indicate that intact import machinery is essential for UPR^mt^-induced lifespan extension. Taken together, our findings suggest that mitochondrial import machinery is transcriptionally upregulated upon UPR^mt^ induction, and import capacity is elevated, thereby facilitating the restoration of mitochondrial proteostasis and inducing lifespan extension (Fig. 7 c).

## Discussion

Metazoans have evolved various defense mechanisms to protect themselves against the detrimental consequences of stress and aging. Many of the stress-responsive mechanisms require altering the composition of their proteomes. This remodeling often includes enhancing the networks of stress-responsive proteins and chaperones, which are targeted for specific subcellular compartments or organelles that are stressed.

It has been proposed that the alteration of mitochondrial import plays a role in the induction of mitochondrial UPR (Nargund et al., 2012). However, the regulation of mitochondrial import upon stress has not been investigated in depth. In this work, we revealed the augmentation of mitochondrial protein import as a downstream effect of the mitochondrial stress response. This upregulation of import is specifically associated with the induction of UPR^mt^, instead of being a generic secondary effect of delayed aging or prolonged lifespan. We did not observe upregulation of the mitochondrial membrane potential that correlates with the import competency. In fact, we found that in spite of decreased membrane potential, the UPR^mt^ resulted in increased import activity. Taken together, our findings indicate that the enhancement of import is mediated by transcriptional regulation of the mitochondrial import machinery, and our in vitro biochemical assays reveal increased import competency of these mitochondria. Intriguingly, the efficiency of mitochondrial import serves as an active mechanism of increased longevity upon the activation of the UPR^mt^.

It was previously proposed that mitochondrial import deteriorates upon mitochondrial stress and thereby excludes ATFS-1 from mitochondria, allowing it to enter the nucleus to induce the expression of downstream stress-response genes (Nargund et al., 2012). Surprisingly, we found that the UPR^mt^-dependent upregulation of import is true not only for general import, but also for ATFS-1, the import deficiency–dependent messenger of stress. Our findings raise the question of whether and how the UPR^mt^ is maintained upon upregulation of the UPR^mt^. It was recently revealed that mitochondrial stress during larval development induces chromatin changes that are perpetuated into adulthood and make up a critical part of the UPR^mt^ (Merkwirth et al., 2016; Tian et al., 2016). Accordingly, UPR^mt^ induced by a transient deficiency in import may be sufficient to self-sustain the downstream effects, including the prolonged upregulation of import. In fact, it was found that the expression of ATFS-1 itself is upregulated upon induction of the UPR^mt^ (Nargund et al., 2012), suggesting that once the UPR^mt^ is activated, nuclear ATFS-1 might be kept at a higher level despite the recovery of mitochondrial import efficiency.

Notably, mitochondria exhibit a high level of heterogeneity within cells (Kuznetsov and Margreiter, 2009). It is conceivable that among the large population of mitochondria within a cell, some might remain at a low-import status and constantly send stress signal to the nucleus, whereas the rescuing proteins, once made, are sent to a relatively healthy subpopulation of mitochondria or are used in the genesis and assembly of a new cohort of mitochondria. Therefore, the higher level of import efficiency upon UPR^mt^ in the in vitro import assay may be due to a fraction of mitochondria being healthier and more resilient due to UPR^mt^ activation and able to be better preserved in the extraction process. In the future, it will be imperative to monitor the mitochondrial import of individual mitochondria in vivo during the aging process and under conditions of UPR^mt^ induction.

Recently, Schäfer and colleagues developed a proteomics approach to quantify mitochondrial protein import (Schäfer et al., 2022). In particular, they used cell-wide and mitochondria-selective proteomics analysis to measure the translation or uptake of mitochondrial proteins, respectively. They found a global reduction of mitochondrial protein uptake when carbonyl cyanide *m*-chlorophenyl hydrazone (CCCP) was used to inhibit mitochondrial membrane potential. Of note, the mitochondrial protein profile showed a more selective and less robust change upon the treatment of MitoBloCK-6, which mostly impairs the import of intermembrane space proteins. This is consistent with the previous finding that different triggers of mitochondrial stresses result in very different transcriptional and proteomic responses (Quirós et al., 2017). In the future, it would be very interesting to employ this paradigm to investigate the regulation of mitochondrial import upon different types of mitochondrial stress, including the UPR^mt^.

## Materials and methods

### Strains

CB4037 glp-1(e2141) III, CF512 *fer-15(b26) II, fem-1(hc17ts)* IV, CF596 *daf-2(mu150) III; fer-15(b26); fem-1(hc17ts)*, SJ4100 *(zcIs13[hsp-6p::gfp])*, N2 wild-type strains were obtained from the Caenorhabditis Genetics Center (Minneapolis, MN). AGD1484 (*hsp-6p::hsp-6::gfp*) strain was generated with the Mos1-mediated single copy insertion (MosSCI) technique.

### RNAi feeding

Worms were grown from the hatch on HT115 *Escherichia coli* containing an empty vector control or expressing double-stranded RNA. RNAi strains were from the Vidal library if present or the Ahringer library if absent from the Vidal library.

### Import assay

pGEM4-su9(1-69)-DHFR plasmid was a gift from Dr. Thomas Langer (Teichmann et al., 1996). Fusion protein su9-DHFR was transcribed, translated, and biotinylated in a single reaction with the TnT SP6 Quick Coupled Transcription/Translation System (L2080; Promega) and Transcend tRNA (L5016; Promega). Mitochondria extraction was made from synchronized worms at the designated age in mitochondria extraction buffer (5 mM Tris-HCl, pH 7.4, 210 mM mannitol, 70 mM sucrose, and 0.1 mM EDTA). Protease inhibitor (Protease Inhibitor Cocktail Set III, EDTA-Free; 539134; Calbiochem) was used at 1:1,000. Worms were mechanically homogenized with Dura-Grind Stainless Steel Dounce Tissue Grinder (357572; Wheaton), and mitochondria were isolated via differential centrifugation. Mitochondria pellets were resuspended in buffer C (20 mM potassium Hepes and 0.6 M sorbitol). Protein concentration was measured using BCA Protein Assay Kit (23225; Pierce), and the same amount was used in each import reaction.

Mitochondrial import assay was performed as previously described (Ryan et al., 2001). Briefly, the precursor protein was incubated with 50 μg fresh mitochondria extraction in import buffer (50 mM Hepes, 1 mg/ml fatty acid–free BSA, 0.6 M sorbitol, 50 mM KCl, 10 mM Mg_2_Cl, 2.5 mM EDTA, and 2 mM KH_2_PO_4_, pH 7.4) containing 2 mM ATP and an ATP regeneration system (0.2 mg/ml creatine kinase [10127566001; Roche], 5 mM creatine phosphate [10621714001; Sigma-Aldrich], 4 mM malate, and 4 mM succinate) for 10–45 min at 25°C with gentle shaking. Import assay with a single time point was performed with a 30-min incubation time unless otherwise noted. Membrane potential (ΔΨ) was disrupted with valinomycin in control. Mitochondria were subsequently treated with proteinase K to degrade preproteins that are attached to the surface of the mitochondria. Mitochondria were spun down and resuspended in mitochondria extraction buffer. SDS (6×) loading buffer was added to each sample. Samples were heated at 95°C for 5 min and resolved by NuPAGE Bis-Tris mini gels, followed by Western blot with DHFR antibody. Import efficiency was quantified by measuring the mature imported protein as detected by the DHFR antibody in ImageStudio (LiCor). Signal intensity was normalized against the signal intensity of control treatment with 30-min incubation time unless otherwise noted. Data were analyzed using unpaired *t* test (two-tailed) with Prism (GraphPad4).

### Subcellular fractionation

Synchronized worms were lysed, and mitochondria were isolated via differential centrifugation as previously described (Daniele et al., 2016). Supernatant before and after the centrifugation for mitochondria (12,000 *g*, 10 min) were kept as total and cytosolic portion, respectively. Samples were heated at 95°C for 5 min and resolved by Bio-Rad Mini-PROTEAN TGX precast protein gels, followed by Western blot. Proteins of interest were detected with Azure Sapphire Biomolecular Imager and quantified with AzureSpot.

### Immunostaining

*hsp-6p::hsp-6::gfp* animals were bleach synchronized and treated with *tomm-20* and/or *cco-1* RNAi. Particularly, for *tomm-20 cco-1* double RNAi, the animals were grown on bacteria growing *tomm-20* double-stranded RNA (dsRNA) from hatch and for the first day of development, then transferred to bacteria expressing *tomm-20* and *cco-1* dsRNA and grown for 2 d; for *cco-1* RNAi, animals were grown on empty vector control RNAi plates from hatch and for the first day of development, then transferred to bacteria expressing *cco-1* dsRNA (diluted to 1:1 ratio with bacteria containing the empty RNAi vector alone to match the strength in double RNAi treatment) for 2 d; for *tomm-20* RNAi, animals were grown on bacteria expressing *tomm-20* dsRNA from hatch and for the first day of development, and for the next 2 d, the *tomm-20* dsRNA was diluted to 1:1 ratio with bacteria containing the empty RNAi vector alone to match the strength in double RNAi treatment; for the EV control, animals were grown on empty vector control RNAi plate from hatch for 3 d. 3 d after bleaching, animals at young adult/day 1 stage were cut open at the posterior end to expose the intestine for fixation and immunostaining. Animals were then fixed with 1% PFA and stained with GFP antibody (Rabbit) and ATP5a antibody (Mouse), followed by corresponding secondary antibodies (Alexa Fluor 488 donkey anti-rabbit, Alexa Fluor 647 chicken anti-mouse). Animals were mounted on slides with 20 mg/ml *n*-propyl gallate in 70% glycerol. Images were acquired with NIS-Elements on a Nikon A1 confocal microscope, with objective lens, PlanApo VC100×/1.4 oil, at room temperature.

Fluorescent images were analyzed with Fiji. In particular, integrated density was measured for total GFP and GFP within the region of interest set with the staining of mitochondrial marker ATP5a. The ratio between mitochondrial GFP and total GFP was calculated.

### Fumarase assay

For *cco-1* single RNAi, *glp-1* animals were bleach synchronized and grown at 25°C on bacteria expressing *cco-1* dsRNA (or the empty vector control) until the first day of adulthood. For sequential double RNAi, glp-1 animals were bleach synchronized and grown at 25°C overnight on bacteria expressing *tomm-20* dsRNA (or the empty vector control). Animals were then transferred to grow on bacteria expressing both *cco-1* and *tomm-20* dsRNA (or *cco-1* dsRNA diluted to 1:1 ratio with bacteria containing the empty RNAi vector alone to match the strength in double RNAi treatment) until the first day of adulthood. Different fractions were separated via differential centrifugation. Proteins in each fraction were quantified with BCA assay, and an equal amount of protein from each fraction was used in fumarase assay. Fumarase activity was measured with a Colorimetric Assay Kit (MAK206; Sigma-Aldrich). The result was also confirmed with measuring fumarase activity in 100 mM potassium phosphate buffer with L-malic acid (malate) as substrate. Absorption at 240 nm was measured with a Tecan infinite 200 plate reader.

### Statistical analysis

Statistical analysis was performed as described in the figure legends. Data distribution was assumed to be normal but was not formally tested.

### Quantitative PCR (qPCR)

Total RNA was harvested from worms at the early adult day 1 stage using TRIzol LS Reagent (Life Technologies). After freezing and thawing three times, RNA was purified on RNeasy mini columns (Qiagen), and cDNA was synthesized using the QuantiTect Reverse Transcription kit (Qiagen). SybrGreen RT-qPCR experiments were performed as described in the manual using QuantStudio 6 Flex Real-Time PCR System. Internal controls used a geometric mean of *cdc-42*, *pmp-3*, and *Y45F10D.4*. Experiments were repeated three times. Primers used for qPCR were hsp-6 forward 5′-CAA​ACT​CCT​GTG​TCA​GTA​TCA​TGG​AAG​G-3′; hsp-6 reverse 5′-GCT​GGC​TTT​GAC​AAT​CTT​GTA​TGG​AAC​G-3′; tomm-20 forward 5′-CGG​CTA​CTG​CAT​TTA​CTT​CGA-3′; tomm-20 reverse 5′-TCA​TTG​CCT​GCT​GCA​GCT​GGA-3′; tomm-22 forward 5′-CGA​CTT​CGT​TCA​GCA​GTT​CAT-3′; tomm-22 reverse 5′-GCG​ATC​AAT​GAC​GTT​GTA​GAT​A-3′; tomm-40 forward 5′-AGC​TCG​TGA​TGT​CTT​CCC​AAC-3′; tomm-40 reverse 5′-TCC​AAA​TCG​GTA​TCC​GGT​GTT-3′; timm-17B.1 forward 5′-GAT​TGT​TGT​CTT​GTC​GCC​ATC​C-3′; timm-17B.1 reverse 5′-ATC​ACC​TTT​GGT​CCT​GAA​CGG-3′; timm-23 forward 5′-AGT​GCC​GGA​ATG​AAC​TTC​TC-3′; timm-23 reverse 5′-GTT​GAT​CCA​AGG​CGA​GGA​C-3′; tin-44 forward 5′-GGG​ATA​CGA​TTA​ACT​CGG​ACA-3′; tin-44 reverse 5′-CTG​CAT​TCG​AGC​TTT​CAA​CTG-3′; mppa-1 forward 5′-CGA​TTT​TGT​GAC​TGT​TGG​CGT-3′; mppa-1 reverse 5′-GCT​TGA​GAA​CGA​TTC​CGA​TGA-3′; mppb-1 forward 5′-GCA​CAA​GTT​CAG​CCG​AAA​TCA-3′; mppb-1 reverse 5′-TTC​TCA​TTC​TCG​TAG​CGA​CTG-3′; cdc-42 forward 5′-AGG​AAC​GTC​TTC​CTT​GTC​TCC-3′; cdc-42 reverse 5′-GGA​CAT​AGA​AAG​AAA​AAC​ACA​GTC​AC-3′; pmp-3 forward 5′-CGG​TGT​TAA​AAC​TCA​CTG​GAG​A-3′; pmp-3 reverse 5′-TCG​TGA​AGT​TCC​ATA​ACA​CGA-3′; Y45F10D.4 forward 5′-AAG​CGT​CGG​AAC​AGG​AAT​C-3′; and Y45F10D.4 reverse 5′-TTT​TTC​CGT​TAT​CGT​CGA​CTC-3′.

### Antibodies

A polyclonal rabbit antibody to TIMM-23 was generated against the synthesized polypeptide of amino acids 95–230 and affinity-purified (ABClonal Science). Other antibodies and probes used for Western blot were as follows: anti-DHFR antibody (D1067; Sigma-Aldrich), anti-HSP-6 antibody (ADI-SPS-825; Enzo Life Science); anti-NDUF3 (17D95) antibody (ab14711; Abcam); anti-GFP antibody (NB100-62622; Novus); anti-ATP5a antibody (ab14748; Abcam); IRDye 680CW donkey anti-mouse IgG (H + L; 926-68072; LI-COR); IRDye 680LT donkey anti-rabbit IgG (H + L; 926-68023; LI-COR); Alexa Fluor 647 chicken anti-mouse A21463, and Alexa Fluor 488 donkey anti-sheep A11051.

### TMRE staining

TMRE staining was performed according to the previous study (Cho et al., 2017). TMRE was dissolved in DMSO at a concentration of 50 µM and added into fresh bacteria culture at a final concentration of 0.1 µM before seeding the plates. Worms were synchronized by egg bleach and grown on *E. coli* HT115 for RNAi from the hatch and transferred to RNAi plates containing TMRE at the L3/L4 stage. Worms were imaged after growing overnight on TMRE plates. TMRE staining was quantified with ImageJ. CCCP was dissolved in DMSO at a concentration of 10 mM and added into bacteria culture at a final concentration of 50 µM before seeding the plates. Images were acquired with Leica Application Suite (LAS) using Fluorescent Stereo Microscope Leica M205, with objective lens, PlanApo 5.0× LWD (10447243; Leica), and camera, Leica DFC3000 G, at room temperature.

### Lifespan analysis

Lifespan experiments were performed with glp-1(e2141) III worms at 25°C as previously described (Dillin et al., 2002). Worms were synchronized by egg bleach and grown on *E. coli* HT115 for RNAi from the hatch. Worms were scored every second day. Prism 6 software was used for statistical analysis. Log-rank (Mantel–Cox) method was used to determine the significant difference.

### Online supplemental material

Fig. S1 shows that *mrps-5* knockdown promotes mitochondrial import. Fig. S2 shows that Ponceau S staining shows protein at equivalent levels. Fig. S3 shows that *spg-7* knockdown enhances the transcription of import machinery components. Fig. S4 shows that *daf-2* mutation or *daf-2* knockdown does not enhance mitochondrial import.

## Supplementary Material

SourceData F1 is the source file for Fig. 1.

SourceData F2 is the source file for Fig. 2.

SourceData F3 is the source file for Fig. 3.

SourceData F4 is the source file for Fig. 4.

SourceData F5 is the source file for Fig. 5.

SourceData F6 is the source file for Fig. 6.

SourceData FS1 is the source file for Fig. S1.

SourceData FS4 is the source file for Fig. S4.
