# Supplementary figures and images for: The UPRmt preserves mitochondrial import to extend lifespan
Source: J Cell Biol. 2022 May 24;221(7):e202201071. doi: 10.1083/jcb.202201071 (PMC9134095; doi:10.1083/jcb.202201071)

Figure 1

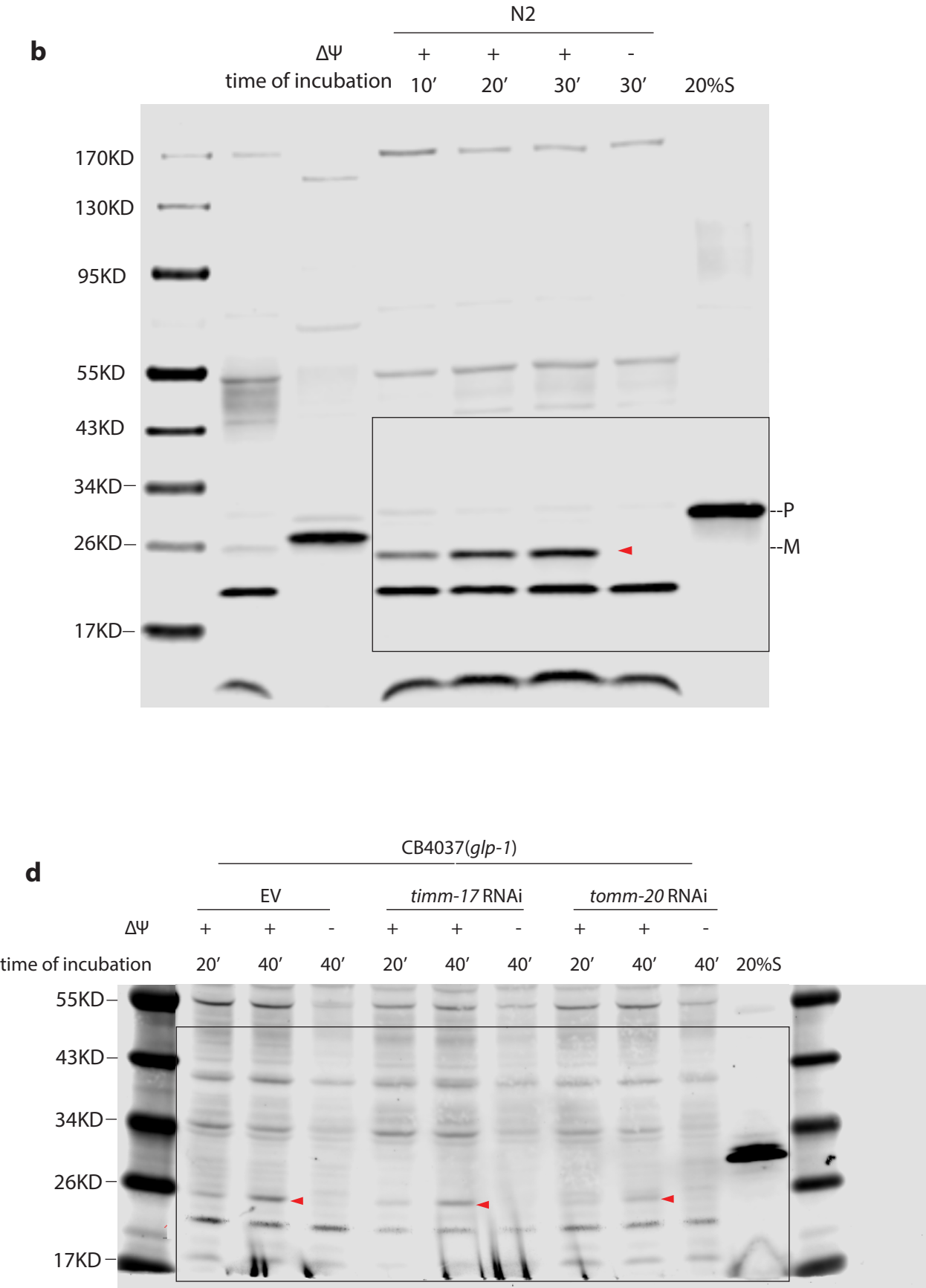

Supplement: SourceData F1 — is the source file for Fig. 1. [file JCB_202201071_SourceDataF1.pdf]

Figure 2

a

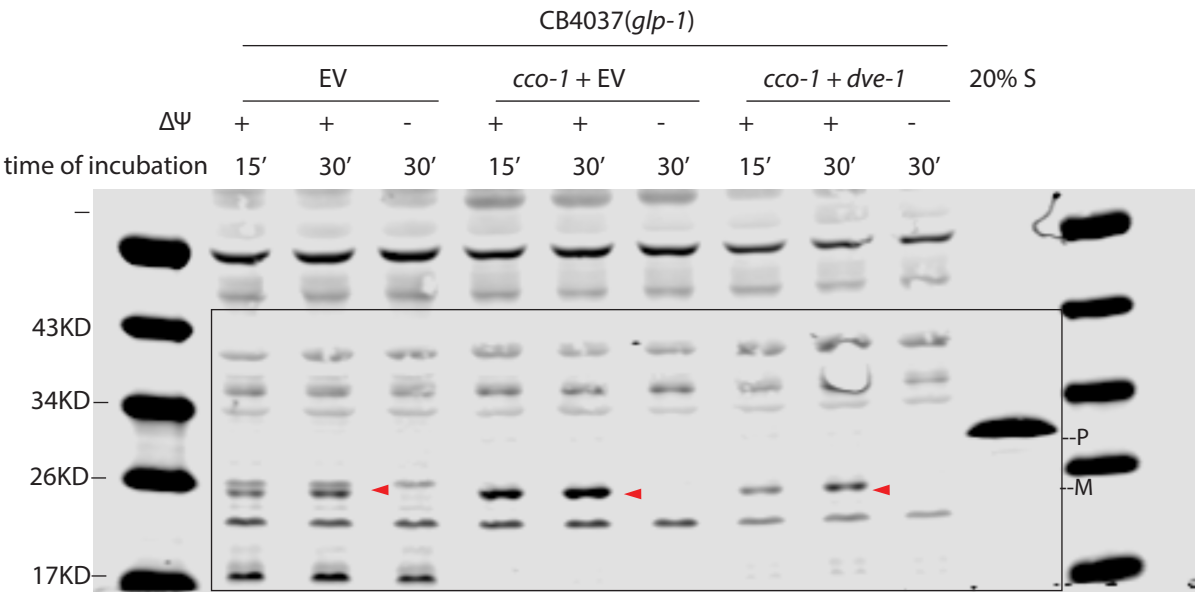

c

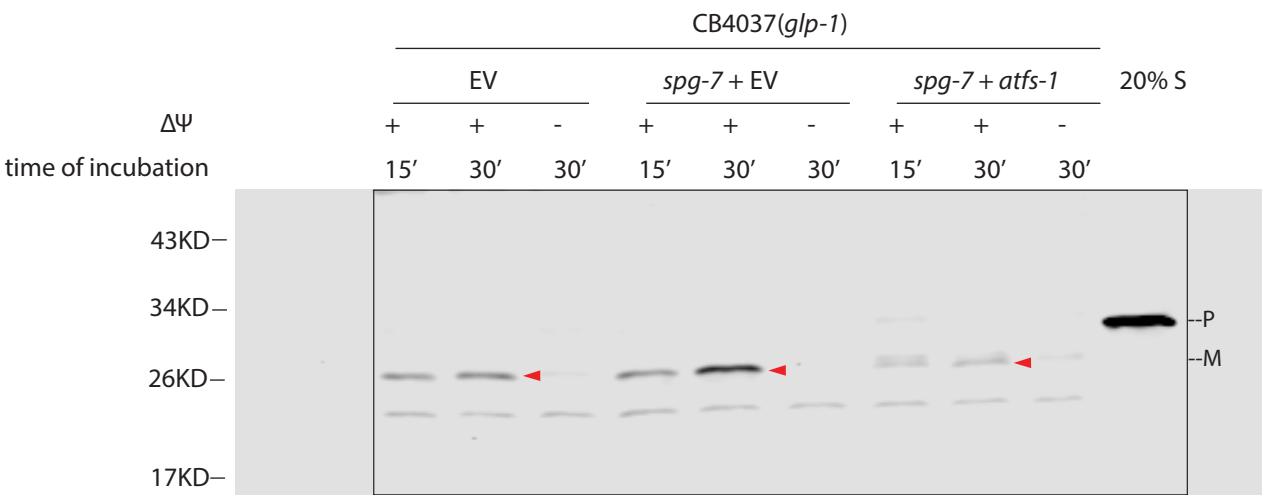

Supplement: SourceData F2 — is the source file for Fig. 2. [file JCB_202201071_SourceDataF2.pdf]

Figure 3

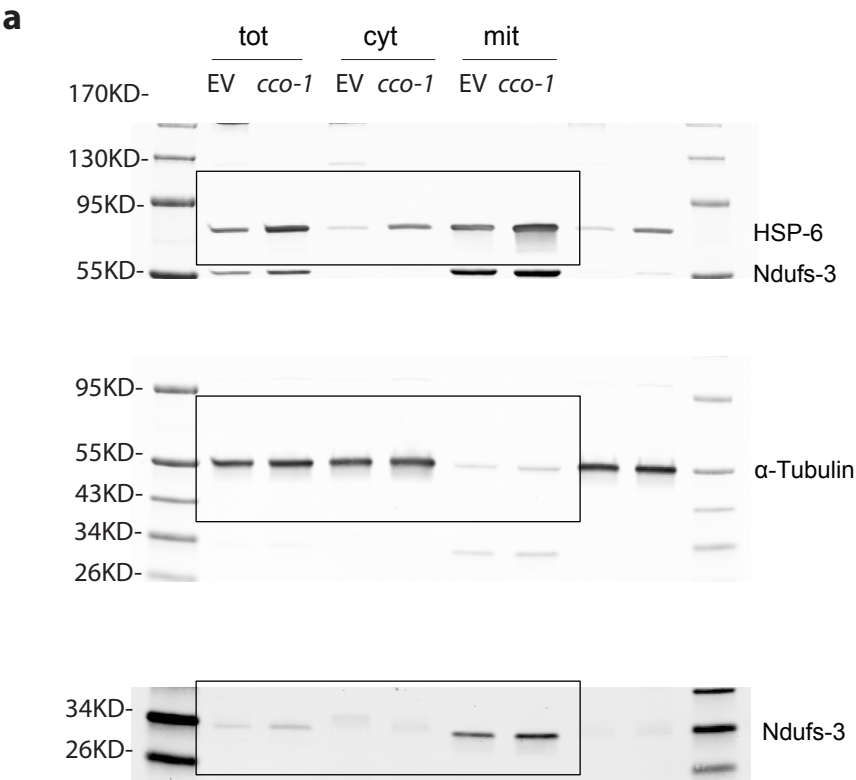

Supplement: SourceData F3 — is the source file for Fig. 3. [file JCB_202201071_SourceDataF3.pdf]

Figure 4

**b**

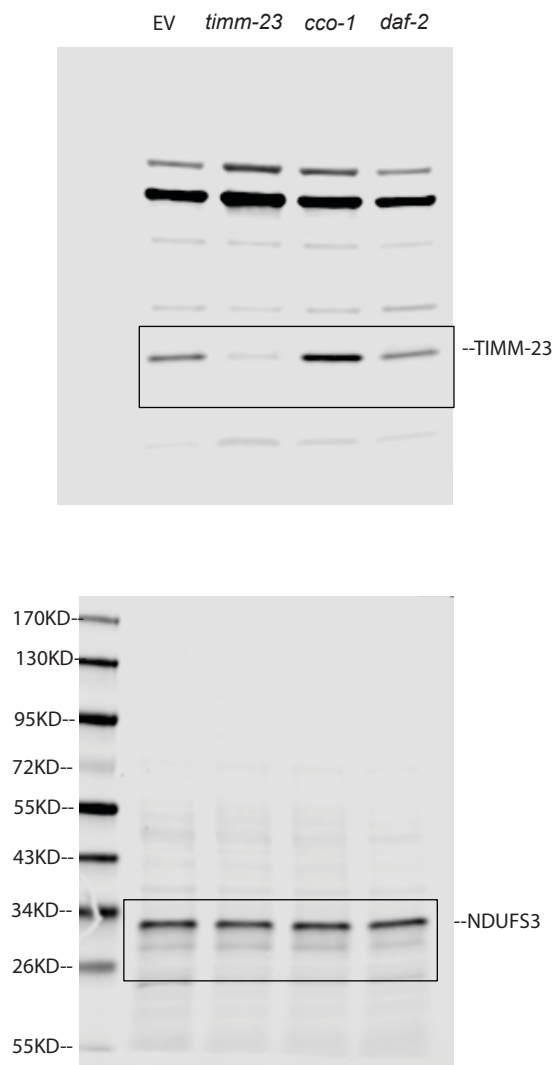

Supplement: SourceData F4 — is the source file for Fig. 4. [file JCB_202201071_SourceDataF4.pdf]

Figure 5

a

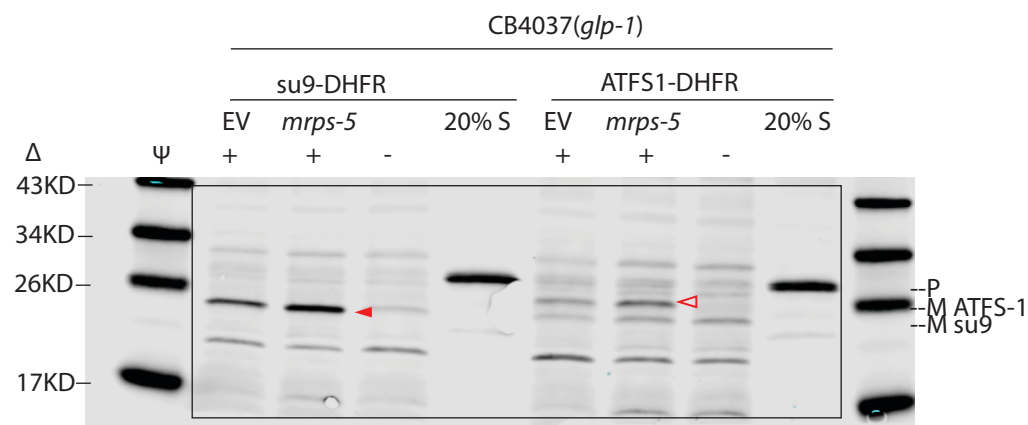

d

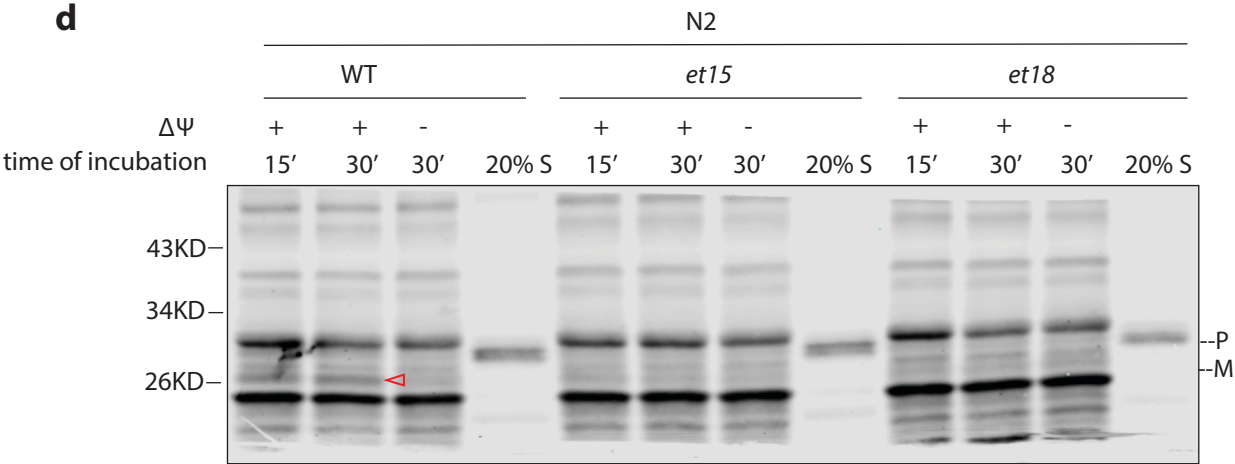

Supplement: SourceData F5 — is the source file for Fig. 5. [file JCB_202201071_SourceDataF5.pdf]

Figure 6

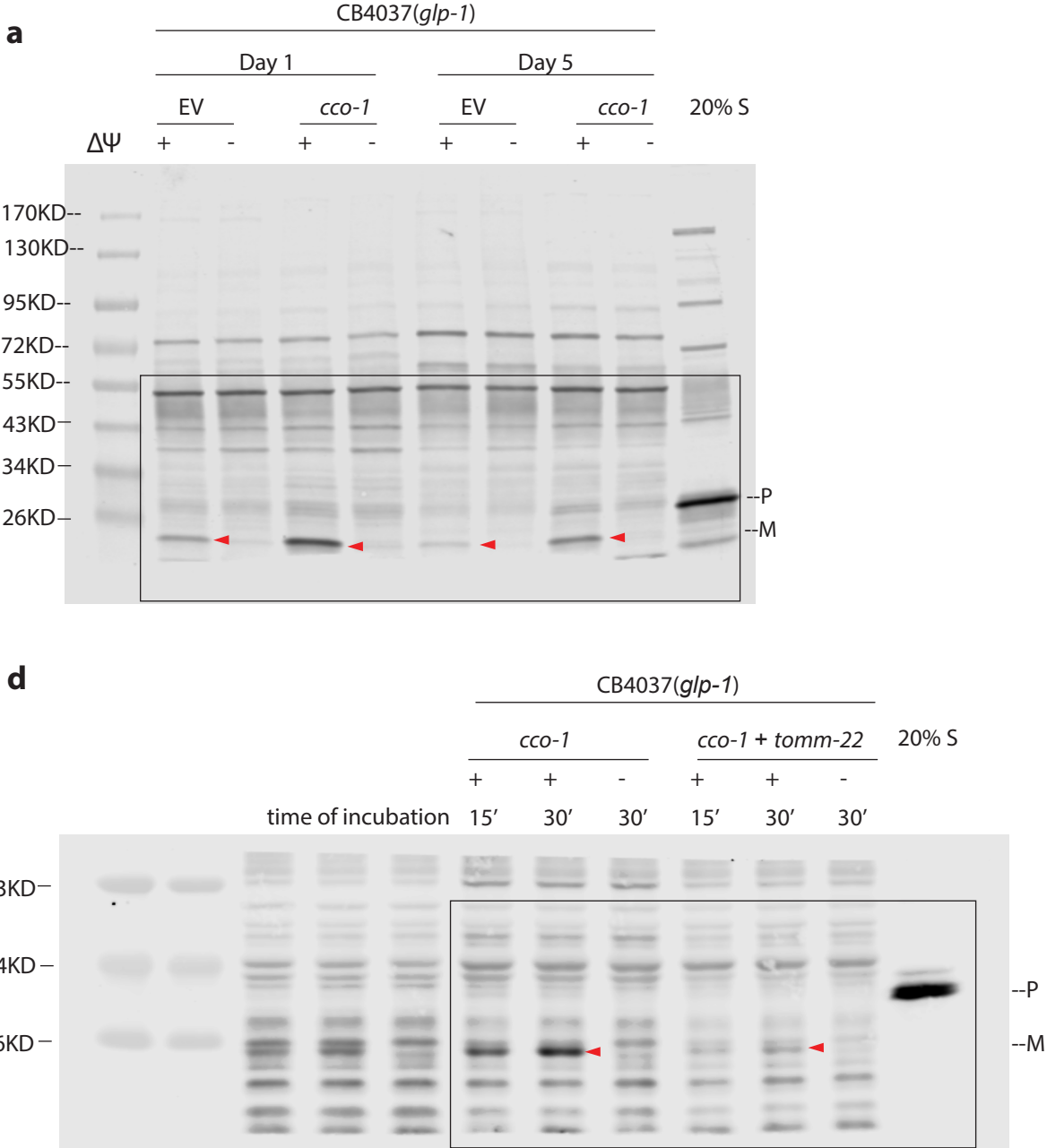

Supplement: SourceData F6 — is the source file for Fig. 6. [file JCB_202201071_SourceDataF6.pdf]

## Supplemental Figure 1

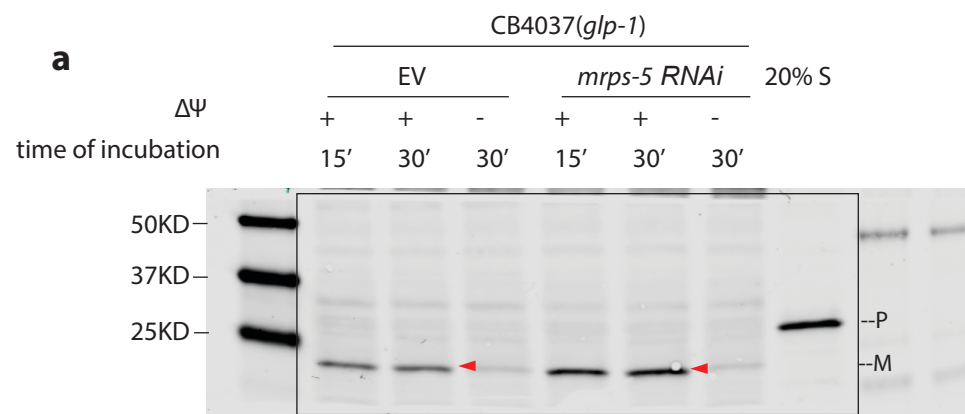

Supplement: SourceData FS1 — is the source file for Fig. S1. [file JCB_202201071_SourceDataFS1.pdf]

# Supplemental Figure 4

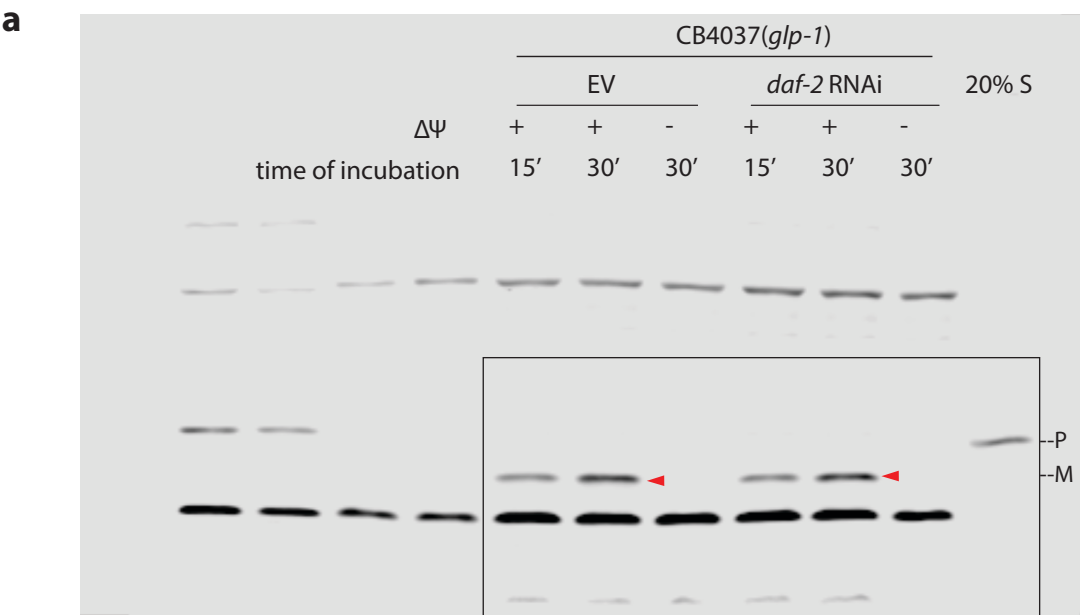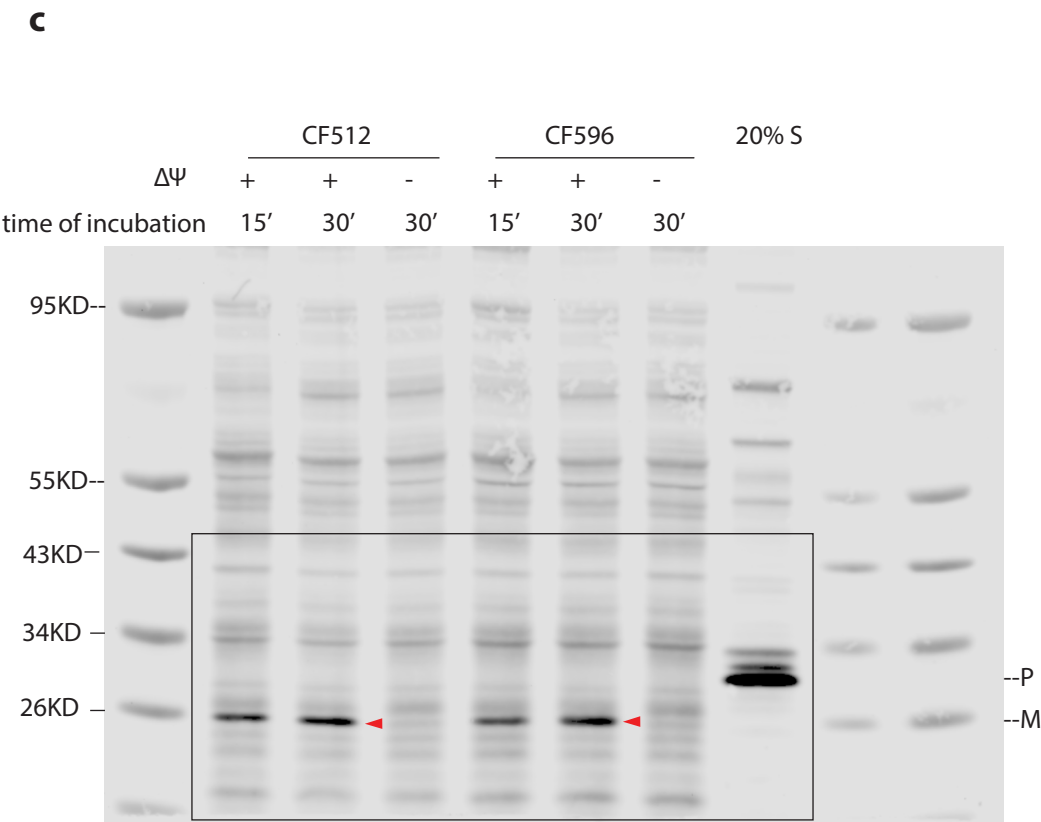

Supplement: SourceData FS4 — is the source file for Fig. S4. [file JCB_202201071_SourceDataFS4.pdf]
